# 3D-printed micrometer-scale wireless magnetic cilia with metachronal programmability

**DOI:** 10.1126/sciadv.adf9462

**Published:** 2023-03-22

**Authors:** Shuaizhong Zhang, Xinghao Hu, Meng Li, Ugur Bozuyuk, Rongjing Zhang, Eylul Suadiye, Jie Han, Fan Wang, Patrick Onck, Metin Sitti

**Affiliations:** ^1^ Physical Intelligence Department, Max Planck Institute for Intelligent Systems, 70569 Stuttgart, Germany.; ^2^ State Key Laboratory of Solidification Processing, Center of Advanced Lubrication and Seal Materials, Northwestern Polytechnical University, Xi'an 710072, China.; ^3^ Central Scientific Facility Materials, Max Planck Institute for Intelligent Systems, 70569 Stuttgart, Germany.; ^4^ State Key Laboratory for Manufacturing Systems Engineering, Xi’an Jiaotong University, Xi’an 710054, China.; ^5^ Zernike Institute for Advanced Materials, University of Groningen, 9747 AG Groningen, Netherlands.; ^6^ Institute for Biomedical Engineering, ETH Zürich, 8092 Zürich, Switzerland.; ^7^ School of Medicine and College of Engineering, Koç University, 34450 Istanbul, Turkey.

## Abstract

Biological cilia play essential roles in self-propulsion, food capture, and cell transportation by performing coordinated metachronal motions. Experimental studies to emulate the biological cilia metachronal coordination are challenging at the micrometer length scale because of current limitations in fabrication methods and materials. We report on the creation of wirelessly actuated magnetic artificial cilia with biocompatibility and metachronal programmability at the micrometer length scale. Each cilium is fabricated by direct laser printing a silk fibroin hydrogel beam affixed to a hard magnetic FePt Janus microparticle. The 3D-printed cilia show stable actuation performance, high temperature resistance, and high mechanical endurance. Programmable metachronal coordination can be achieved by programming the orientation of the identically magnetized FePt Janus microparticles, which enables the generation of versatile microfluidic patterns. Our platform offers an unprecedented solution to create bioinspired microcilia for programmable microfluidic systems, biomedical engineering, and biocompatible implants.

## Teaser

3D-printed biocompatible biological-sized cilia demonstrate good programmability and controlled liquid transporting capacity.

## INTRODUCTION

Cilia are microscopic hair-like cellular protrusions with a typical length between 1 and 30 μm beating at tens of Hertz in the low–Reynolds number (*Re*) regime (*Re* ≪ 1) (*1*, *2*). They are crucial to the survival and health of many biological systems [including human beings (*3*)] by performing essential functions, such as self-propulsion (*4*), food capture (*5*, *6*), self-cleaning (*7*, *8*), cerebrospinal fluid transport (*9*), mucus clearance (*10*), and egg cell delivery (*11*). These intriguing functions have inspired the creation of artificial cilia devices, such as on-chip microfluidic pumps and mixers (*12*, *13*), self-cleaning and antifouling surfaces (*14*, *15*), and droplet and particle manipulation platforms (*16*, *17*). Magnetic artificial cilia (MAC) use magnetic field as a wireless actuating stimulus (*18*–*20*). Comparing with artificial cilia driven by other stimuli (*21*–*25*), MAC can instantaneously respond to external magnetic fields and show robust controllability. Moreover, the magnetic actuation offers a safe interaction between the artificial cilia and the surrounding environment because no temperature increase or chemical changes are introduced. Biological cilia often exhibit a more complex beating pattern of metachronal wave motions, namely, adjacent cilia beat with a certain phase difference (*26*). Numerical simulations have unveiled that the metachronal motions are critical in enhancing the fluid transportation efficiencies of cilia arrays (*27*–*29*). The advances of understanding the importance of metachrony trigger the creations of metachronal artificial cilia (*24*). In literature, there are two common strategies to create metachronal MAC: (i) apply a nonuniform magnetic field to an array of identical MAC (*30*) and (ii) design an array of MAC of varying magnetization directions or mechanical properties so they respond heterogeneously to a uniform magnetic field (*18*–*20*, *31*). The latter strategy is preferable because of the ease of encoding the MAC with complex magnetic profiles, leading to more freedom to program the metachrony with various phase differences (*19*, *20*). Moreover, it usually requires a simpler and cheaper actuation setup (*20*), while the first method needs a cumbersome and expensive actuation system (*30*). The previously reported metachronal MAC either lack the freedom of programming arbitrary phase differences (*18*, *30*, *32*), which leads to constrained functionalities, or they are too large in size (*19*, *20*), which limits their applications in microfluidic devices where fluids (such as reagents and cell culture media) are continuously perfused into channels that are in sizes ranging from tens to hundreds of micrometers (*33*, *34*).

In the context of biomedical applications, MAC arrays can be implanted inside the human body for controlled transportation of biofluids or objects and/or be used to study cells, tissues, and organs in bioengineering (*34*, *35*). One important design consideration for these applications is the biocompatibility of the materials used. However, the biocompatibility of MAC has not been demonstrated so far. Most of the magnetic composites used to fabricate MAC are known to be toxic to cells (*36*, *37*), such as hard magnetic neodymium-iron-boron (NdFeB) and soft magnetic nickel and cobalt. Biocompatible paramagnetic materials, such as iron oxide nanoparticles, however, have low magnetic remanence and low coercivity, which require strong actuation magnetic fields and are not able to retain programmed magnetization profiles (*32*, *38*). Recent studies have shown that the L1_0_ ordered phase of iron-platinum (FePt) is noncytotoxic, nonimmunogenic, and highly biocompatible, while having large magnetic remanence, large coercivity, and good chemical stability (*39*–*42*). Owing to these combined properties, FePt particles/coatings have been proven to be a suitable candidate to create biocompatible magnetic micromachines. Kadiri *et al.* (*40*) demonstrated a facile strategy to fabricate biocompatible FePt nanopropellers for cell transfection by co-depositing Fe and Pt onto silica followed by a single annealing step. Bozuyuk *et al.* (*41*) demonstrated biocompatible FePt microrollers that can move upstream against blood flows under magnetic actuation and are compatible with multimodal high-contrast medical imaging.

“Janus” particles, named after the two-faced Roman god Janus, impart drastically different chemical/physical properties and directionality within a single particle by breaking the surface symmetry (*43*, *44*). Magnetic Janus particles have the unique property of magnetic shape anisotropy, which creates precise magnetic domains with the resolution of the size of a single particle. Recently, we demonstrated the creation of one-dimensional (1D) to 3D programmable magnetic micromachines with an overall size less than 100 μm using the two-photon polymerization (2PP) and magnetic manipulation techniques to selectively link nickel-iron alloy (NiFe) Janus microparticles (JMPs) with gelatin (*45*). Although NiFe is soft magnetic, their magnetization programmability was enabled by the inherent magnetic shape anisotropy of the JMPs. Nevertheless, less magnetic torque could be generated in comparison with hard magnetic materials. FePt-based Janus particles could fulfill both the biocompatibility and magnetic programmability requirements. In the previous work, the soft links made of gelatin were not always robust mechanically because they were sensitive to temperature changes and swell. On the contrary, silk fibroin (SF) is a Food and Drug Administration (FDA)–approved protein-based biopolymer, exhibiting low immunogenicity, excellent biocompatibility, tailorable biodegradability, and, more importantly, robust mechanical properties. SF has been applied for flexible electronics, advanced optics, photonics, and tissue engineering scaffolds (*46*–*50*). 3D printing of SF at micrometer scale has been demonstrated (*31*, *51*). However, the mechanical properties of SF hydrogel at the length scale of less than 100 μm have not been reported yet. The ability to create robust SF structures at such small scales and being able to characterize and tune their mechanical properties would boost their use in future microscopic applications.

Here, we use hard magnetic FePt JMPs and biopolymer SF to 3D print biocompatible and programmable MAC arrays at a length scale close to biological cilia using 2PP in combination with magnetic actuation controls. Magnetic, mechanical, and biological properties of the fabricated cilia were systematically investigated, showing that our cilia were mechanically tunable and robust, biocompatible, and biodegradable. Upon applying a uniform rotating magnetic field of as small as 3 mT, each cilium performed a whip-like reciprocal motion consisting of a slow forward stroke and a fast backward stroke. The difference in the orientation between neighboring FePt JMPs endowed the MAC array with heterogeneous magnetization directions, initiating a metachronal wave within the MAC array when subjected to a uniform rotating magnetic field. We demonstrated the flexibility of our fabrication platform by creating MAC arrays with designated phase differences and various locational arrangements. Although each cilium beats in a reciprocal manner, liquid transportation in the low *Re* regime (*Re* < 0.1) could be fulfilled by breaking the symmetry through attaching a flag-shaped structure on each cilium. We showcased the design and fabrication of MAC arrays to generate translational flows and locally circulating flows. This work could provide an experimental platform for biocompatible and programmable microcilia arrays with a large design space for future potential applications in microfluidic devices and cilia-inspired biomedical soft microrobots.

## RESULTS

### Fabrication of programmable MAC arrays

**Fig. 1.**
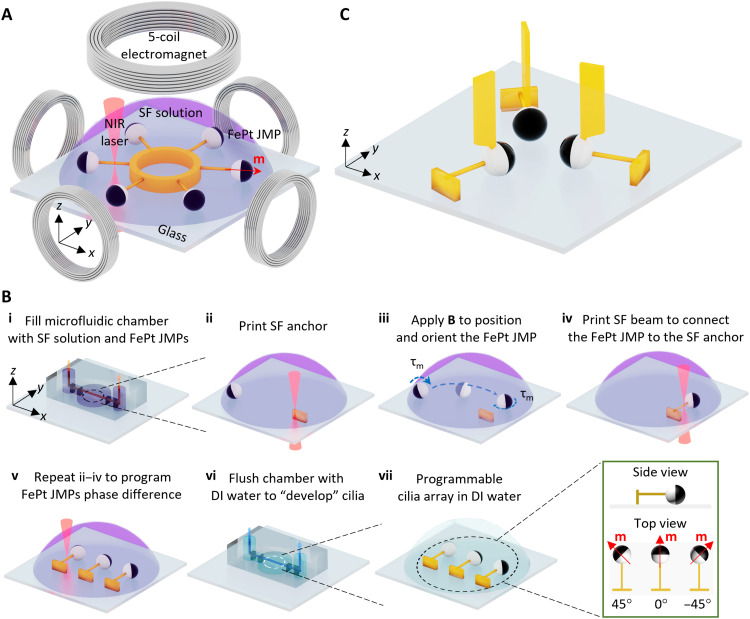
Fabrication of programmable MAC arrays. (**A**) Schematics of the fabrication platform consisting of a five-coil electromagnetic setup integrated inside a 2PP system (3D direct laser writing system) working with near-infrared (NIR) laser (see also fig. S1), showing that a MAC array composed of six cilia in a circular arrangement with a 60° orientation difference (i.e., phase difference) is being printed by cross-linking SF. The integrated electromagnetic setup enables the positioning and orienting of the premagnetized hard magnetic FePt JMPs whose magnetization direction is indicated by the red arrow (see fig. S2 and movie S1). (**B**) Schematics of the fabrication process of a representative MAC array with three cilia in a line with a phase difference of 45° (see movie S2). (**C**) Schematics of one representative programmable MAC array consisting of three cilia facing each other in a triangular configuration (phase difference = 120°) with each cilium containing a 3D flag-shaped additional structure, demonstrating the large design space of the platform. The geometry of the flag-shaped structure is shown in fig. S4. Illustrations are not to scale. Photo credit: Meng Li, Xinghao Hu, and Shuaizhong Zhang, Max Planck Institute for Intelligent Systems.

The programmable MAC arrays were fabricated by 3D printing an SF beam on the transparent half of the FePt JMPs using a commercial 2PP system integrated with a customized electromagnetic actuation setup (Fig. 1A and fig. S1). The integrated magnetic system enabled positioning and orienting the hard magnetic FePt JMPs (movie S1). The particle magnetization direction was along the normal direction of the plane separating the FePt and silica halves (fig. S2). The details of the fabrication process are as follows (Fig. 1B; see also movie S2). First, the mixture of SF hydrogel precursor solution and FePt JMPs (10 μm in diameter) was filled into a sealed polydimethylsiloxane (PDMS) microfluidic chamber (Fig. 1B and fig. S3). Second, a rectangular SF base (width × height × thickness = 10 μm × 10 μm × 5 μm) was direct laser printed on the glass substrate, serving as an anchor for the cilium. Third, by applying an out-of-plane rotating magnetic field, an FePt JMP was rolled on the glass substrate to a target location. Because the magnetic moment (**m**) direction of the FePt JMP tends to align with the direction of the applied magnetic field (**B**), one can adjust the orientation of the FePt JMP by applying an in-plane static magnetic field in the desired direction. Fourth, an SF beam (width × height × length = 1 μm × 1 μm × 10 μm) was printed to connect the FePt JMP and the SF anchor. Fifth, an array of cilia was created by repeating steps (ii) to (iv). For example, one can print three parallel cilia with a 45° phase difference of **m**. Sixth, the 3D-printed structures were “developed” by flushing the microfluidic channel with deionized (DI) water using a syringe pump at a flow rate of 0.5 μl min^−1^ for 20 min. This low flushing flow rate helps maintain the structural integrity of the printed SF beams. Last, the programmed cilia arrays were obtained on a glass substrate submerged in water. Owing to the ease of controlling the positions and orientations of the hard magnetic FePt JMPs, MAC arrays with arbitrary phase difference and locational arrangements could be programmed, such as a circular configuration with six cilia pointing outward (phase difference = 60°, Fig. 1A) and a triangular configuration with three cilia pointing at each other (phase difference = 120°; Fig. 1C). In the latter case, each cilium had an additional flag-shaped structure (fig. S4), which can be easily modified owing to the design flexibility and high 3D printing resolution (~100 nm) of the 2PP system. Note that the fabrication precision of the MAC was mainly affected by the accuracy of the FePt JMP positioning. For the 50 MAC samples that we tested, the SD of the MAC length is 0.37 μm, which is 3.6% of the average value (10.4 μm).

### Magnetic, mechanical, and biological properties of the MAC arrays

**Fig. 2.**
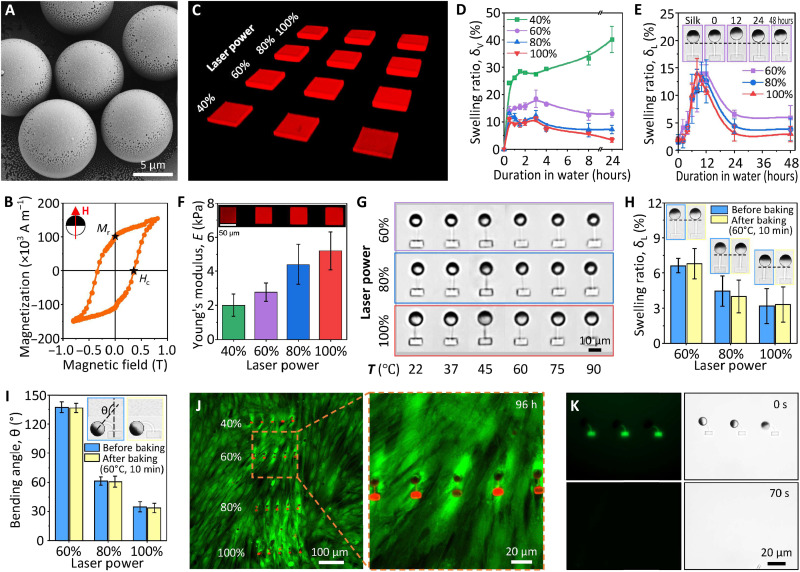
Magnetic, mechanical, and biological property characterization results of the MAC. (**A**) Scanning electron microscopy (SEM) image of the FePt JMPs. (**B**) Magnetic hysteresis loop of the FePt JMPs. The inset shows the relative direction of the FePt JMPs and the applied magnetic field. (**C**) 3D oblique view of the confocal images of the SF blocks. (**D**) Volumetric swelling behavior of the printed SF blocks over time analyzed using the confocal images (see fig. S6). (**E**) Linear swelling behavior of the MAC over time. The insets show the swelling of one cilium printed with a laser power of 60%. (**F**) Young’s modulus of the SF blocks (see fig. S7). (**G**) Top-view optical microscopic images of the MAC under various temperatures, showing no visible shape change. (**H**) Measured linear swelling ratio of the MAC before and after baking at 60°C for 10 min. The insets show the microscopy images of the MAC before and after baking. (**I**) Measured maximal bending angle of the MAC under a uniform rotating magnetic field of 3 mT before and after baking at 60°C for 10 min. The insets show the images of a bending cilium printed with a laser power of 60% before and after baking. (**J**) Fluorescent images of human fibroblast cells after 96 hours cocultured with the MAC, showing no dead cells, which should exhibit a red color. The red dots are the printed SF beams and SF anchors. (**K**) Fluorescent and bright-field microscopic images of the MAC under a flow of protease XIV solution (1 U ml^−1^) at a flow rate of 2 μl min^−1^, showing quick biodegradation of the printed SF. Error bars indicate SDs for *n* ≥ 5 measurements. Photo credit: Shuaizhong Zhang, Meng Li, and Ugur Bozuyuk, Max Planck Institute for Intelligent Systems.

The magnetic, mechanical, and biological properties of the fabricated MAC arrays were characterized systematically. FePt JMPs were fabricated by co-depositing Fe and Pt on a monolayer of silica particles (10 μm in diameter; SiO2-R-SC223; microParticles GmbH) using a molecular beam epitaxy system (see Materials and Methods). The FePt coating was 60 nm thick (*41*) and homogeneous (Fig. 2A and fig. S5). X-ray photoelectron spectroscopy (XPS) survey scan measurements revealed that the thin film had a 37 to 63% (±2%) ratio of Fe and Pt, which is within the range to form L1_0_ phase of FePt (*42*). As shown in Fig. 2B, the FePt JMPs have a coercivity of 350 mT and a remanence of 100 × 10^3^ A m^−1^ which gives each FePt JMP a magnetic moment (**m**) of 9.5 × 10^−13^A·m^2^. The high coercivity prevents the FePt JMPs from being remagnetized by the actuating magnetic fields (less than 10 mT). The not-too-low remanence enables the FePt JMPs to locomote swiftly following a small rotating magnetic field (10 mT). In the meantime, the not-too-large remanence causes less aggregation problems comparing with magnetized NdFeB particles; two FePt JMPs brought to contact can still be separated using magnetic controls (movie S3).

The mechanical properties of the MAC arrays were investigated using SF microstructures fabricated using a series of laser powers with a constant scanning speed of 10,000 μm s^−1^. First, time-dependent swelling was studied through measuring the volumetric change of SF blocks (length × width × height = 50 μm × 50 μm × 18 μm) over time (Fig. 2C and fig. S6; Materials and Methods). After an initial swelling and subsequent shrinking process (except for the samples printed at a laser power of 40%), the 2PP-printed SF structures reached a swelling equilibrium state after being soaked in DI water for about 1 day (Fig. 2D). The volumetric swelling ratio is defined by $\delta_{V}=\frac{V_{t}-V_{o}}{V_{o}}$, where *V_o_* and *V_t_* represent the volume of the SF-blocks immediately after developing and *t* hours after soaking in DI water, respectively. In the final equilibrium state, the volumetric swelling ratio for SF blocks printed with a laser power of 40, 60, 80, and 100% was 40, 13, 7, and 3%, respectively. As expected, a higher laser power results in a lower swelling ratio due to a higher cross-linking density in the SF hydrogel network. During the development, unreacted reagents (photoinitiators) and uncross-linked polypeptide chains diffuse into the surrounding water and the cross-linked hydrogel network takes in water and swells. During the first 10 hours, the hydrogel volume kept increasing. Then, it decreased and reached equilibrium after 24 hours. Similar trend was also observed in literature but the reason is unclear (*52*).

Next, we directly studied the 1D swelling behavior of the printed cilia by measuring the lengths of the cilia (see Materials and Methods). The results depicted in Fig. 2E show that cilia continuously swelled in the first ~10 hours of development, reaching a maximal linear swelling ratio of around 15% for all cases (60, 80, and 100% laser power). The cilia gradually shrunk in the next 10 hours. After approximately 24 hours of soaking in DI water, they finally achieved an equilibrium state of 7, 4, and 3% for 60, 80, and 100% printing power, respectively. The trend is the same as the aforementioned results of SF blocks, although there is a discrepancy in the values. Here, the linear swell ratio is defined by $\delta_{L}=\frac{L_{t}-L_{\mathrm{silk}}}{L_{\mathrm{silk}}}$, where *L*_silk_ and *L_t_* represent the length of the SF beam as printed in SF solution and *t* hours after developing with DI water, respectively. Note the calculated volumetric swelling ratio (1 + δ*_L_*)^3^ using the linear swelling ratio of the SF beams is larger than the directly measured δ*_V_* of the printed SF blocks. This can be explained by a larger surface area/volume ratio of the beam than the block (4.2 μm^−1^ versus 0.14 μm^−1^), which facilitates the substance diffusion and water intake. Therefore, SF beams swelled more rapidly than the SF blocks. Moreover, unlike suspended SF beams, one side of the SF blocks was fixed on the glass substrate; hence, their swelling was restricted to a certain extent.

The Young’s modulus (*E*) of the equilibrated SF blocks (i.e., 1 day soaked in DI water) was measured by nanoindentation using an atomic force microscope (AFM; fig. S7). The results showed that the fabricated SF structures had a Young’s modulus of between 1 and 10 kPa, the same order as soft biological tissues such as human liver. A higher laser power gives a larger Young’s modulus, making it possible to control the mechanical properties of the printed SF structures (Fig. 2F). The viscoelasticity of the SF hydrogels was characterized via dynamic oscillation, showing that the SF hydrogel was elasticity-dominated under a wide range of frequencies (fig. S8).

As the working temperature of microfluidic cilia may exceed the room temperature, for example, in organ-on-a-chip applications where human or animal tissues and/or organs are cultured and studied in a microfluidic chip (*34*, *35*), we further investigated the mechanical stability of the MAC under elevated temperatures ranging from 22°C (room temperature) to 90°C. All MAC fabricated with a laser power ranging from 60 to 100% at a constant printing speed of 10,000 μm s^−1^ showed no visible structural deterioration (Fig. 2G). The conclusion stands for MAC printed using different printing speeds as well (fig. S9). To further confirm their mechanical stability, we checked the swelling behavior and actuation performance of MAC arrays before and after baking at 60°C for 10 min under a rotating uniform magnetic field of 3 mT. The results depicted in Fig. 2 (H and I) showed negligible change in the geometry and actuation performance before and after baking. The resilience to temperature change shown by these SF-based MAC makes sample preparation and actuation more repeatable and dependable.

Biological properties of the fabricated MAC arrays were characterized in terms of biocompatibility and biodegradability, as they are the common prerequisites for the feasibility of cilia in biomedical applications. To demonstrate the biocompatibility of the MAC arrays, we cultured them with a human fibroblast cell line. We seeded the fibroblast cells on top of the MAC and observed their viability after 72 hours of culturing using live-dead staining. Fluorescent images showed that the cells were completely viable and exhibited healthy spindle-shaped morphology near and around the MAC arrays (Fig. 2J). Moreover, the printed SF structures can be degraded on demand using enzymes. The SF components of the MAC arrays degraded within 70 s under a flow of protease XIV solution (1 U ml^−1^) at a flow rate of 2 μl min^−1^ at room temperature (Fig. 2K and movie S4). Note that the enzyme concentration that we used in this study is orders of magnitude higher than physiological levels. In physiological environment, the silk hydrogel could take weeks or even months to degrade depending on the degree of hydrogel cross-linking, types of enzymes, environmental pH and temperature, etc. This biodegradability enhances the impact of SF materials on applications where eliminating existing structures and/or rebuilding new designs are favorable. In addition, the degradation of SF links turns the anchored FePt JMPs into surface microrollers, which can locomote and perform other functions as reported before (*41*, *53*). In phosphate-buffered saline (PBS), MAC experienced geometrical shrinkage instead of swelling as in DI water (fig. S10). This is because the ion-rich environment outside the hydrogel created an osmotic gradient that drives water out. In addition, under the same fabrication and actuation conditions, MAC exhibited an approximately 30% larger bending angle in PBS than in DI water.

### Actuation of the MAC arrays

**Fig. 3.**
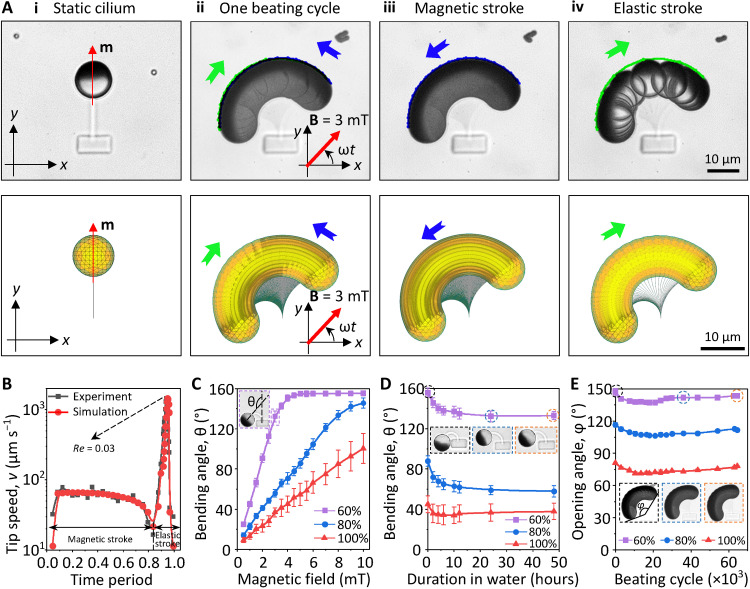
Magnetic actuation of the MAC using rotating uniform magnetic fields. (**A**) Experimental (top) and numerically simulated (bottom) cilium motion at 1 Hz under a uniform magnetic field of 3 mT rotating in the CCW direction, showing a 2D reciprocal motion (see also movie S5). (i) Bottom view of one static cilium with the red arrow indicating the magnetic moment (**m**) direction of the FePt JMP. (ii to iv) Time-lapse images of the cilium motion during one beating cycle at 1 Hz. The images are composed of image sequences with an identical time interval of 0.002 s. The blue and green arrows indicate the direction of the magnetic stroke and the elastic stroke, respectively. (**B**) Cilia tip speed during one beating cycle at 1 Hz obtained experimentally and numerically. (**C**) Maximal bending angle θ of the MAC as a function of the amplitude of the applied magnetic field. The inset shows the definition of θ. (**D**) Maximal bending angle θ of the MAC printed over time under a uniform magnetic field of 3 mT rotating in the CCW direction. The insets show the bending of one cilium at times 0, 24, and 48 hours after developing with DI water, respectively. (**E**) Opening angle ϕ of the MAC as a function of their beating cycles, showing only slight change in the motion. The insets show the time-lapse image of a cilium during one beating cycle at 5 Hz at cycle number 1, 36,000, and 64,500, respectively. The definition of ϕ is indicated in the inset. The MAC reported in (A) and (B) are fabricated with a laser power of 60%. Error bars indicate SDs for *n* ≥ 5 measurements. Photo credit: Shuaizhong Zhang and Rongjing Zhang, Max Planck Institute for Intelligent Systems.

The actuation experiments of the MAC arrays were performed using equilibrated 1-day-old samples after developing with DI water. Using four current-controlled electromagnetic coils, we actuated the MAC arrays under an in-plane uniform rotating magnetic field of 3 mT in the counterclockwise (CCW) direction. Each cilium performed a 2D whipping motion consisting of two strokes: (i) a “magnetic stroke” (indicated by the blue line) when the cilium bent CCW mostly following the applied magnetic field and accumulated elastic energy in the beam until the phase lag between the FePt JMP magnetization direction (**m**) and the applied field direction (**B**) reached the maximum and (ii) an “elastic stroke” (indicated by the green line) when the cilium released the accumulated elastic energy and whipped back clockwise (CW) to its original position (Fig. 3A and movie S5). The whipping motion has been reported experimentally and numerically by many previous studies (*18*, *20*, *30*, *32*, *54*), and it resulted from a rotational buckling instability that is induced by the nonlinear relations between internal elastic stress, hydrodynamic forces, and exerted external magnetic torques. Classically, ciliary beating strokes are termed effective/power stroke and recovery stroke for a nonreciprocal motion. However, this is not applicable to our case because of the reciprocal nature of our cilia motion unveiled by recording the strokes with a high-speed camera and confirmed numerically (Fig. 3A, ii). With a CCW rotating magnetic field at 1 Hz, the cilium moved slowly during the magnetic stroke with a tip speed in the order of 100 μm/s and snapped back rapidly during the elastic stroke with a tip speed of 1500 μm/s (Fig. 3B). Here, the local *Re* is defined by *Re* = ρν*L*/η, in which ρ is the density of the fluid, *v* is the speed of the cilia tip, *L* is the length of the cilia (approximately 20 μm), and η is the dynamic viscosity of the fluid. For water at room temperature, the local *Re* during elastic stroke is 0.03. At large *Re* regimes, as shown by Zhang *et al.* (*32*) (*Re* ~ 100), reciprocal cilia motion can generate sufficient net water flow, but at the low *Re* regime in our case, viscous effects dominate inertial effects and 2D reciprocal motion is unable to generate a net flow.

The maximum MAC bending angle, θ, was measured as a function of the amplitude of the applied rotating magnetic field. As shown in Fig. 3C, the bending angle of the cilia increased linearly with the amplitude of the applied magnetic field until the FePt JMP reached the silk anchor at 160°. The increase of the bending angle with the magnetic field is because of the increased magnetic torque **τ** = **m** × **B** (where **m** is the magnetic moment of a cilium and **B** is the applied magnetic field). Because of their smaller elastic modulus, the cilia printed with a lower laser power bent more under the same magnetic field (Fig. 2F). As a result of the swelling and equilibrium of the printed SF hydrogel beam, the bending angle showed a decreasing trend for the first ~24 hours of developing in water as shown in Fig. 3D. After that, the bending angle was stable (for at least another 24 hours). Durability and mechanical stability of materials facilitates their ease of use. We magnetically actuated MAC at 5 Hz for ~4 hours continuously, which corresponds for over 65,000 beating cycles. All tested cilia maintained good 2D whipping motions with only slight variation in the opening angle (ϕ) (Fig. 3E). We did not observe MAC damage after 4 hours of operation; only out of preventing our electromagnetic coil from overheating did we stop the actuation. Moreover, as shown in Fig. 3D, our MAC can maintain their actuation property after at least 50 hours after fabrication. These results show that the SF beams are operated in their elastic deformation range with minimum material deterioration, which demonstrates the actuation robustness of our cilia.

### Programmable metachrony

The types of metachronal waves are defined by the relationship between the wave propagating direction and the effective stroke direction. Because our cilia beat in a reciprocal fashion, the traditional definitions are not applicable to define which stroke is the effective stroke. However, to make the discussion easier, we regarded the magnetic stroke as the effective stroke hereafter. Symplectic metachrony indicates that the wave propagating direction and the effective stroke direction are the same; antiplectic metachrony indicates the opposite, and diaplectic metachrony indicates perpendicular. More specifically, for diaplectic metachrony, dexioplectic metachrony and laeoplectic metachrony specify that the angular difference between the effective stroke direction and the wave propagating direction is 90° and −90°, respectively (*55*, *56*).

**Fig. 4.**
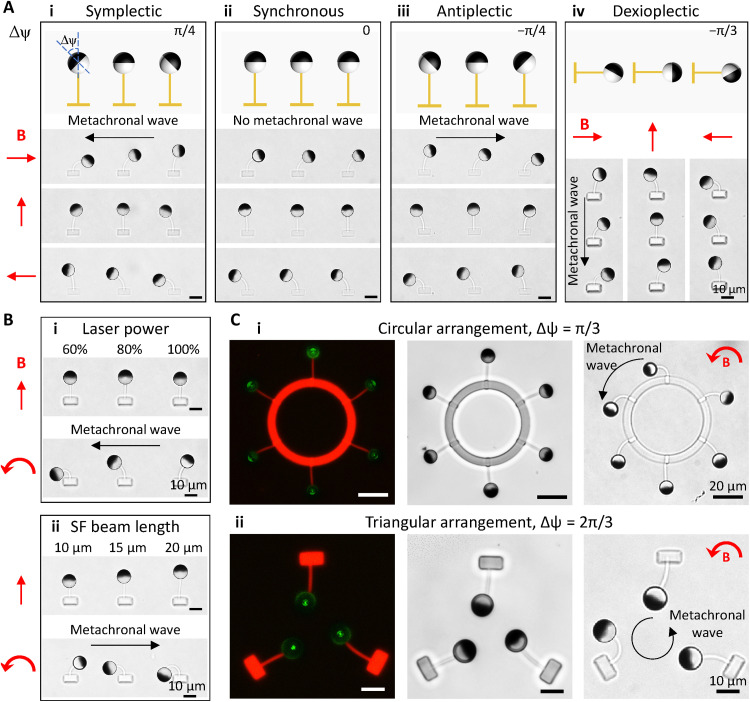
Programmable metachronal MAC array demonstrations. (**A**) Programmable metachrony by changing the orientation of the FePt JMPs and the arrangement of the MAC array (see movie S6). Δψ indicates the orientation difference of the FePt JMPs between neighboring cilia. The black arrows indicate the direction of the propagating metachronal wave. (**B**) Programmable metachrony by changing the mechanical properties (i) and the geometry (ii) of the MAC while keeping the orientation of the FePt JMPs the same (see movie S7). The black arrows indicate the direction of the propagating metachronal wave. (**C**) 2D programmable metachrony with the fluorescent microscopic images (left) showing the SF structures and the bright-field microscopic images (right) showing the orientation of the FePt JMPs and the metachronal motion of the MAC arrays (see movie S8). All the MAC shown here, except for [(B), i], are printed with a laser power of 60%. The uniform rotating magnetic field is kept at 3 mT. Photo credit: Shuaizhong Zhang, Max Planck Institute for Intelligent Systems.

By programming the orientation difference, Δψ, between adjacent FePt JMPs in the cilia array, we were able to create programmable metachrony at a size close to natural cilia with arbitrary phase differences and arrangements (movie S6). When the cilia beams were parallel to each other and Δψ > 0 (i.e., from the top view, the FePt JMP on the left-hand side orients more in the CCW direction), the cilia array performed a symplectic metachronal motion (Fig. 4A, i). Obviously, when Δψ = 0, the cilia array beat in synchronization (Fig. 4A, ii). When Δψ < 0, the cilia array performed an antiplectic metachronal motion (Fig. 4A, iii). Diaplectic metachrony could be created by arranging the cilia in a head-to-tail manner while maintaining Δψ ≠ 0 (Fig. 4A, iv). Note that the metachrony could not be changed by altering the rotating direction of the applied magnetic field (movie S6). To validate the accuracy of the MAC fabrication process, we measured the experimental phase difference ΔΦ of seven representative examples, namely, Δψ = −π/3, −π/4, −π/6, 0, π/6, π/4, and π/3. Here, the phase difference ΔΦ is measured by $\text{Δ}\text{Φ}=\frac{\text{Δ}t}{T}\;\cdot \;2\pi \;[\text{Δ}t\in (-T,T)]$, where Δ*t* is the time lag between neighboring cilia when each cilium reaches its upright position during the magnetic stroke and *T* is the beating period. The results demonstrated that the deviation between the designed Δψ and experimental ΔΦ is within 5% (fig. S11).

Metachrony could also be created by keeping Δψ = 0 while varying the mechanical properties and/or the geometries of the SF beams such as by changing the printing power and the beam length (Fig. 4B and movie S7). In addition, by taking advantage of the large printing freedom, we could print MAC with designed 2D arrangements. For example, we created MAC array in a circular configuration with cilia tips facing outward (Δψ = π/3) and in a triangular configuration with cilia tips pointing inward (Δψ = 2π/3) (Fig. 4C and movie S8).

### Microscale fluid transporting capability

**Fig. 5.**
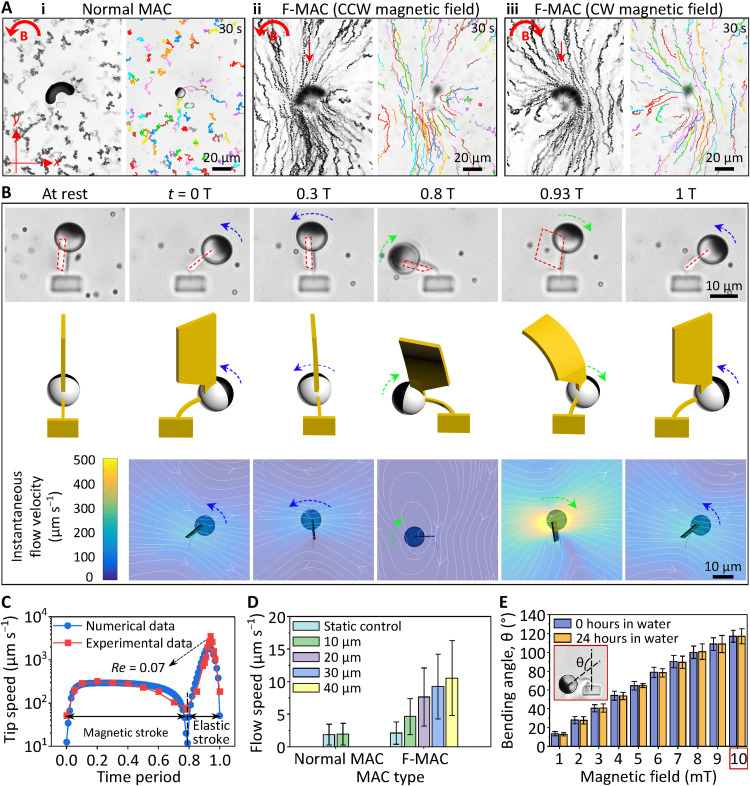
Fluid transporting capability of a single cilium. (**A**) Time-lapse (left) and particle tracking (right) images of the generated flow by a single cilium over a period of 30 s (see movie S10). The overlapping images are composed of 150 consecutive images of 30-s videos. The observation layers for (i) to (iii) were 10, 40, and 40 μm above the substrate, respectively. The red arrows in (ii) and (iii) indicate the flow direction. The results of the static control experiments are shown in fig. S12. (**B**) Bottom-view microscopic images (top), oblique-view schematics (middle), and top-view modeling results (bottom) of the F-MAC beating at 5 Hz, showing the deflection of the flag-shaped structure during the magnetic (blue arrows) and elastic (green arrows) strokes and the induced instantaneous flow (white lines in the modeling results). The red dashed lines indicate the projected flag on the substrate plane. See also fig. S13 and movie S9. (**C**) F-MAC tip speed during a single beating cycle at 5 Hz. (**D**) Measured flow speed generated by both a single normal MAC and an F-MAC. (**E**) Maximal bending angle θ of the F-MAC as a function of the amplitude of the applied magnetic field immediately and after 24 hours of developing with DI water. The inset shows the microscopic image of an F-MAC bending at 10 mT and the definition of θ. All the MAC beams and the flag-shaped structures shown here are printed with a laser power of 60 and 40%, respectively. The applied uniform rotating magnetic field is 3 and 6 mT for the normal MAC and the F-MAC in (A) to (D), respectively. Error bars indicate SDs for *n* ≥ 5 measurements. Photo credit: Shuaizhong Zhang and Rongjing Zhang, Max Planck Institute for Intelligent Systems.

Although different from the working principles of the metachronal waves in biological systems, which are commonly believed to be the hydrodynamic interactions (*57*, *58*) and distal couplings among the cilia bands (*59*), our system resembles their biological counterparts in terms of metachronal motion and effective fluid transportation. However, no net flow was induced by a single cilium after a full beating cycle (Fig. 5A, i) because of the reciprocal motion carried out at low *Re*. To generate a net flow, one needs to break the reciprocal nature of the MAC motion. To do that, we printed a flag-shaped structure on the transparent silica half of the FePt JMP (Fig. 5B and fig. S4). Hereafter, the MAC with the additional flag-shaped structure is named F-MAC. Under a rotating uniform magnetic field (6 mT in the CCW direction), while the F-MAC body (SF beam and FePt JMP) still beats reciprocally, the additional flag moved nonreciprocally. The motion was both observed experimentally and confirmed numerically (Fig. 5B, fig. S13, and movie S9). This nonreciprocity was derived from the drastically different beating speed of the F-MAC body between the elastic stroke and the magnetic stroke (Fig. 5C). The 15-times-faster beating speed during the elastic speed led to stronger hydrodynamic drag forces on the flag structures, resulting in larger flag bending and thus smaller sweeping area and less fluid propulsion (note S1). The bending of the flag can be described by the dimensionless Sperm number, *Sp*, which defines the ratio of viscous-to-elastic forces acting on each cilium (*60*). *Sp* is defined as *L*[8π^2^η*f*/*EI*]^1/4^, where *L* is the length of the flag (30 μm), η is the viscosity of the liquid (1 mPa·s for water), *f* is the beating frequency of the flag, *E* is the Young’s modulus of the flag (~2 kPa according to Fig. 2F), and *I* = *tw*^3^/12 is the area moment of inertia of the flag with *t* and *w* as the thickness (2 μm) and width (10 μm) of the flag, respectively. The calculated *Sp* for 5-Hz magnetic beating is approximately 1, a balance between the viscous forces and the elastic forces, indicating that negligible flag shape change is induced during the magnetic stroke. Because the cilia move much faster during the elastic stroke, the equivalent beating frequency is larger than the actuating frequency. Thus, the *Sp* during the elastic strokes is considerably larger than 1, indicating the dominance of the viscous forces that induce shape changes to the flag during the elastic strokes. Thus, a nonreciprocal motion is generated.

As shown in Fig. 5A (ii), the nonreciprocal motion of the F-MAC that was attributed to the difference of the flag sweeping areas (note S1) could generate a net flow in the negative *y* direction (parallel to the long axis of the SF beam; see also movie S10). The flow direction did not change from changing the rotating direction of the applied magnetic field [Fig. 5A (iii) and movie S10]. Note that the maximal local *Re* of the F-MAC was 0.07 (Fig. 5C), indicating that the F-MAC is operating in the regime where viscous effects dominate over inertial effects. We analyzed the flow quantitatively using a customized Python code (see Materials and Methods). The speed of the generated net flow by a single F-MAC beating at 5 Hz was around 10 μm s^−1^ at a height level of 40 μm above the glass substrate (which is the top edge of the flag; Fig. 5D). The bending behavior of the F-MAC was also studied as a function of the applied magnetic field. As shown in Fig. 5E, the bending angle increased almost linearly with the magnetic field, reaching a bending angle of 125° at 10 mT. After 24 hours of soaking in water, the bending angles did not show a statistical difference between all magnetic field amplitudes, demonstrating a good mechanical stability of the printed structures. Moreover, high-speed videos show that our F-MAC could follow the applied magnetic field of up to 100 Hz (the highest frequency our setups can achieve), and no stepping-out was observed (movie S11). Although the bending angle of the F-MAC at 100 Hz obviously decreased, it can be remedied by increasing the magnitude of the applied magnetic field.

**Fig. 6.**
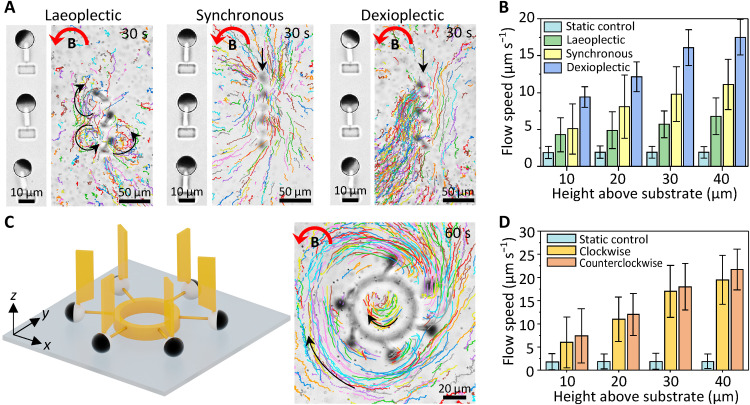
Programmable flow patterns. (**A**) Bottom-view optical microscopic images (left) and particle tracking results (right) of the F-MAC arrays with different metachronal configurations. The phase difference of the laeoplectic and dexioplectic metachrony is π/3 and −π/3, respectively. The particle tracking images are composed of 150 images over a 30-s period. The black arrows indicate the flow direction, and the red arrows mark the rotating direction of a 6-mT uniform magnetic field (see movie S12). (**B**) Measured flow speed generated by laeoplectic, synchronous, and dexioplectic F-MAC arrays at different heights above the substrate. (**C**) Schematic of the circular F-MAC arrangement (left) and the time-lapse particle tracking results (right). The particle tracking image is composed of 300 images over a 60-s period. The black arrows indicate the flow direction, and the red arrow marks the rotating direction of a 6-mT uniform magnetic field. The results under a rotating magnetic field in the CW direction can be found in fig. S14 (see movie S13). (**D**) Measured flow speeds generated by the circular F-MAC array at different heights above the substrate under different rotating directions of the magnetic field. All the MAC beams and the flag-shaped structures shown here are printed with a laser power of 60 and 40%, respectively. Error bars indicate SDs for *n* ≥ 5 measurements. Photo credit: Shuaizhong Zhang, Max Planck Institute for Intelligent Systems.

Here, we demonstrated the microfluidic transportation of the F-MAC array by creating well-controlled complex flow patterns such as local circulating and vortical flows. These flow patterns are inspired by the feeding function of cilia in coral reefs (*61*), and they have potentials to be applied in cell transportation and trapping and mixing flows for biological and chemical analysis (*62*). First, we studied the impact of metachrony on the generated flow patterns. The F-MAC arrays were arranged vertically with a pitch of 40 μm. Figure 6A shows that both synchronous and dexioplectic motion could generate translational flows while laeoplectic motion only induced local chaotic flows. This is probably caused by the so-called shielding effect of neighboring cilia: The flow induced by each cilium is obstructed by the neighboring cilia (*20*, *24*, *63*). The F-MAC with a laeoplectic metachrony shows larger intercilia shielding effect, which leads to local chaotic flows indicated by the chaotic movement of the tracer particles (Fig. 6A). Quantitative analyses revealed that dexioplectic metachrony enhanced the fluidic flow by 150%, while the laeoplectic metachrony actually decreased the flow comparing with the synchronous motion (Fig. 6B). Note that as the net flow direction generated by a single F-MAC is along the negative *y* direction, based on the common definition of the effective stroke in the same direction with the generated flow (*55*, *56*), the laeoplectic metachrony and the dexioplectic metachrony shown here are equivalent to the antiplectic metachrony and the symplectic metachrony, respectively. To avoid confusions with the aforementioned definitions in the “Programmable metachrony” section, we will keep our metachrony definition consistent here.

More complex flow patterns could be created by rationally arranging the F-MAC array. For example, when we arranged the F-MAC in a circular configuration with a phase shift of π/3, a circulating flow outside the cilia array could be generated simultaneously with a mixing flow inside the SF circle (Fig. 6C). At a height level of 40 μm above the glass substrate, the outer flow rate reached 25 μm s^−1^ under a 6-mT rotating field as opposed to a background drifting flow of ~3 μm s^−1^ under no applied field (Fig. 6D). Another example is the inward mixing flow generated by a triangularly configured 3-MAC array (fig. S15), which can find applications, for example, for particle/cell trapping and sample mixing. Vast possibilities could be explored in the future leveraging the programmability of individual cilium and the 3D printing flexibility for various local arrangements.

## DISCUSSION

We have developed wirelessly actuated programmable microfluidic cilia that are composed of FePt JMPs and SF hydrogels at the length scale close to biological cilia. FePt and SF rendered our cilia system biocompatible, allowing its potential applications for biomedical and health care devices. SF also added biodegradability and tunable mechanical characteristics to our design. The high remanence of FePt JMPs in synergy with the low stiffness of SF led to large actuation deformation of our cilia under an external magnetic field of less than 10 mT. In addition, the superior mechanical stability of SF contributed to the robustness of our design over time and under elevated temperatures. Moreover, by adjusting the orientation of the identically magnetized hard FePt JMPs individually, we were able to create a programmable metachrony with controllable phase differences. The metachrony programmability was based on the orientational difference of the FePt JMPs between adjacent cilia, enabling wave propagation along the cilia array under a global rotating magnetic field. Both linear 1D arrangements (including symplectic, antiplectic, and diaplectic metachrony) and 2D arrangements (triangular and circular) could be created. Theoretically, we can fabricate 1D cilia arrays with arbitrary phase differences. However, technically, because of the generation of air bubbles when connecting the SF beam to the opaque FePt halves of the FePt JMPs, only phases ranging from −90° to +90° can be created using the current method. One could extend this range through modifying the fabrication process, such as by printing a transparent assisting ring on the FePt JMPs before connecting the SF beam to the FePt part (*45*). In addition to changing the orientation of the FePt JMPs, metachrony could also be created by introducing variations in the mechanical properties (by tuning, for example, the laser power and/or the laser scanning speed) and/or the geometries (such as the length-to-width ratio) of the neighboring SF beams.

2PP technology enabled the fabrication of sophisticated 3D architectures, yielding nonreciprocal cilia motion and designated cilia arrangements to generate both translational and complex net microfluidic flows. The nonreciprocal motion of the F-MAC, observed experimentally and confirmed numerically, was realized by taking advantage of the huge difference between the moving speeds during the two beating strokes (magnetic and elastic strokes). We found that the dexioplectic metachrony enhanced the fluid transportation by 150% while the laeoplectic metachrony inhibited the flow comparing with the synchronous motion. Previous studies have shown that both metachronies enhance liquid transportation, a result from the interplay of cilia pitch, wave speed, and channel geometry (*2*). The discrepancy between our observation and previous studies can be attributed to the different underlying mechanisms of the nonreciprocal motion: Our cilia have the additional flag structure with reciprocal beating motion, while natural cilia have a simple geometry but with nonreciprocal beating trajectories.

For future work, our method can be used to create MAC with nonreciprocal motion by linking multiple FePt JMPs in a head-to-tail fashion to form the cilia body. The magnetic and actuation programmability arises from the different orientations of neighboring FePt JMPs. One could study their fluid transporting performance with various cilia pitches and metachronal wavelengths in non-Newtonian liquids such as blood and mucus. In addition, we also envisage the realization of cell/particle manipulation (such as transporting, trapping, and repelling) using our cilia by designing starfish larva–inspired cilia arrays (*6*). The current 2D arrangement of FePt JMPs can also be improved to be 3D with the help of sacrificial layers (*31*). With the help of a sacrificial mold featured with microwells (*64*), one could speed up the fabrication process by locating the Janus particles batch by batch instead of one by one, which is currently the most time-consuming step in our fabrication process.

We envision that our robust biocompatible and biodegradable cilia array could encode unprecedented functionalities into artificial cilia at the micrometer length scale to boost their applications in microfluidics, biomedical engineering, and functional surfaces. In addition, cilia in human bodies such as brains, lungs, windpipes (or trachea), and fallopian tubes play very important roles such as mucus propulsion and fluid and egg cell transportation. Our biocompatible MAC could be fabricated on conformable substrates (such as SF thin films) in the future and be implanted to manipulate particles/cells and perform sensing tasks (*1*, *2*).

## MATERIALS AND METHODS

### Fabrication platform of the MAC

The fabrication platform of the MAC consists of a commercially available 2PP-based direct laser writing system (Photonic Professional, Nanoscribe GmbH, Germany) and a 5-coil electromagnetic coil setup (fig. S1) (*45*). A 63× 1.4 numerical aperture oil-immersion objective (Carl Zeiss AG, Germany) was used for 3D microprinting the MAC. A refractive index matching oil (Immersol 518 f; Carl Zeiss Microscopy GmbH) was applied onto the objective to successfully detect the SF solution–substrate interface. The laser power of the 2PP system was 50 mW, which produced a printing power of 20, 30, 40, and 50 mW at a laser intensity of 40, 60, 80, and 100%, respectively. Unless otherwise specified, the laser scanning speed was kept constant at 10,000 μm s^−1^.

### Fabrication of the PDMS microfluidic chip

The microfluidic chip was fabricated using a molding process with PDMS [10:1 (w/w) ratio of the monomer to the cross-linking agent] as precursor materials. The master mold was fabricated by standard photolithography. The PDMS chip, punched with two holes of 1 mm in diameter as inlet and outlet, was bonded to a clean cover glass with dimensions of 12 mm by 12 mm (Marienfeld Superior, Paul Marienfeld GmbH & Co. KG) by oxygen plasma–treating both the PDMS chip and the cover glass, followed by baking in an oven at 90°C for 2 hours. After filling the chamber with the mixture of SF solution and FePt JMPs, the PDMS chip was covered with a thin film of PDMS with thickness of 100 μm after injecting the mixture of SF solution and FePt JMPs to prevent evaporation.

### Fabrication and characterization of the FePt JMPs

The FePt JMPs were fabricated by co-depositing Fe and Pt on a monolayer of nonporous silica (SiO_2_) particles (10 μm in diameter; SiO2-R-SC223; microParticles GmbH) using a custom-built molecular beam epitaxy system under an ultrahigh vacuum condition (≈2 × 10^−9^ to 5 × 10^−9^ mbar) (*41*). Monolayers of the silica particle were prepared using the drop-casting method on an oxygen plasma–treated Si wafer. Fe was evaporated from an effusion cell, and Pt was evaporated from an electron beam evaporator. Both processes were manually controlled to achieve desired deposition rates, which can be measured by a quartz balance. Therefore, an atomic composition ratio of 37 to 63% of Fe and Pt was achieved, which is within the ratio range of the L1_0_ phase of FePt (*42*). During the deposition, the substrate holder was constantly rotating to have a homogenous deposition of FePt thin film of 60 nm. This thickness in combination with the prescribed Fe-Pt ratio enables good magnetic properties while causing less aggregation problems (*41*).

Energy-dispersive x-ray and XPS measurements were conducted with a Thermo Fisher Scientific Theta Probe system for angle-resolved x-ray photoelectron spectroscopy. Magnetic characterizations were performed with a VSM (MicroSense, EZ7, Lowell, MA). These characterizations were done using the as-fabricated FePt JMPs on the Si wafer. The FePt JMPs used for fabricating the MAC were magnetized along the interface normal direction with a 1.8-T magnetic field when they were still on the Si wafer (fig. S2A). After magnetization, the FePt JMPs were released from the Si wafer by ultrasonication in DI water. There were approximately 1000 FePt JMPs per microliter in the final solution.

### Preparation of the SF precursor

SF was regenerated from *Bombyx mori* silkworm cocoons following the established protocols (*65*).

1) Ten grams of cocoons was cut into small pieces and soaked in 4 liters of boiling 0.02 M sodium carbonate/DI water solution for 30 min. This step is to remove the sericin glue that binds the SF fibers together.

2) After degumming, the SF fibers were soaked in 4 liters of DI water for 20 min, and this step was repeated two more times to fully remove dissolved sericin. Note that SF is not water soluble at this point, so after rinsing and soaking, one can just handle the fiber bundles as normal solid.

3) The rinsed SF fibers were taken out of water and laid flat on a clean surface to be fully dried in air at room temperature overnight.

4) The dried SF fibers were dissolved in 9.3 M lithium bromide (LiBr)/DI water solution in a glass beaker. To reach a concentration of 20% w/v, for example, 20 ml of 9.3 M LiBr solution was added onto 4 g of dried SF fibers, which were tightly packed in the glass beaker.

5) Then, the beaker was covered with aluminum foil and kept in an oven at 60°C for 2 hours to allow the SF fibers to dissolve completely.

6) The sample was then dialyzed in 4 liters of DI water for 48 hours with eight changes of water to remove the LiBr and obtain an SF aqueous solution. Water changing was more frequent at earlier times because the solution contains a higher concentration of LiBr at the beginning of the dialysis. For example, we started dialysis at time 0 hours and changed water at time 1, 3, 6, 21 (overnight), 25, 30, and 45 hours (overnight) and collect the solution inside the dialysis tubing at time 48 hours.

7) To remove any solid residues, the SF aqueous solution was centrifuged at 8000 rpm for 20 min at 4°C. This step was repeated until no more visible impurities were presented in the solution.

8) The final SF aqueous solution had a concentration around 6% w/v with a batch-to-batch variation. The exact concentration can be determined by drying a known volume of the solution and then weighing the dried film. The SF solution was stored in a closed tube in a 4°C refrigerator to elongate its shelf time.

The SF solution used for fabricating the MAC was diluted to 5% w/v by adding DI water. Before use, it was filtered using a syringe filter with a pore size of 5 μm to remove impurities. Tris(2,2′-bipyridyl)ruthenium(II) chloride hexahydrate (Ru) and sodium persulfate (SPS) (Sigma-Aldrich) were added into SF solution to make it photocrosslinkable. First, 0.05 M Ru stock solution and 0.05 M SPS stock solution were prepared separately. Then, in 100 μl of 5% w/v SF solution, 2 μl of Ru stock solution was first added and mixed, and 4 μl of SPS solution was then added and mixed. Last, after sonicating the FePt JMPs solution for 20 s, 2 μl was added to the SF mixture. The final concentrations of Ru, SPS, and FePt JMPs in the precursor ink are ~1 mM, ~2 mM, and 20 μl^−1^, respectively. The whole preparation process was conducted in an environment without ultraviolet (UV) light. The precursor ink was used immediately after preparation.

### SF swelling characterization using confocal fluorescent microscopy

The tested samples (length × width × height = 50 μm × 50 μm × 18 μm) were 2PP-printed on a glass slide using various laser powers under a constant scanning speed of 10,000 μm s^−1^. For each printing laser power, three identical hydrogel blocks were printed using the same precursor composition as that for fabricating the MAC. The samples were immersed in DI water and immediately observed under an inverted confocal microscope (Leica SP8 confocal microscope, Wetzlar, Germany) with a 20× objective lens. The confocal imaging was conducted using exciting laser of 522 nm, *z* stack range of 30 μm, and *z* step of 1 μm. Conventionally, to calculate object volume from 3D images, one sets a brightness threshold to generate bi-value images, separating the object from the background. The threshold method is sensitive to fluorescent intensity, which can be affected by laser power, imaging gain, postprocessing, and so on; a small change of the set threshold value can yield markedly different volume values. In our case, the hydrogel samples of different printing laser powers have different fluorescence values, and their fluorescence intensity decreases overtime. Thus, setting one threshold cannot obtain comparable results between different laser power and different time duration. After some exploration with the software, we eventually used an unsharp mask filter with maximum strength (100), maximum gamma value (10), and a high threshold value (253) to separate hydrogel blocks from the background and exclude all the areas of lower contrast (background). This works for the 3D images of all samples with different fluorescence intensities. The obtained convex volume values are stable under different laser powers, imaging settings, and various hydrogel fluorescent intensities and prevented human-introduced errors as in threshold-deciding. Among all the calculated parameters, we chose the convex volume to represent the volume of each hydrogel block. The original volume was measured immediately after developing the printed hydrogels in DI water, annotated as time 0. We kept monitoring the volumetric change for the following 100 hours, but as the fluorescent intensity of the SF hydrogel became weaker as soaking time passed, the images acquired after 24 hours had such a low signal-to-noise ratio that the software could not identify the true block boundaries. Therefore, we only measured the volume of the SF blocks for the first 24 hours.

### SF Young’s modulus characterization using AFM nanoindentation

The SF samples used for characterizing the Young’s modulus were printed using the same parameters as that for the SF swelling characterization. The as-printed samples were thoroughly rinsed with DI water and followed with soaking in DI water for 24 hours to reach hydrogel equilibrium before measurements. The AFM nanoindentation measurements were conducted using a commercial AFM microscope (NanoWizard 4, JPK Instruments) with both the AFM cantilever and the hydrogel samples immersed in DI water for the entire time. The AFM cantilever (CP-CONT-BSG-B-5, NanoAndMore, GmbH) has a 10-μm-diameter spherical indenter attached to the tip. The cantilever was calibrated before each measurement. Specifically, the sensitivity (how much deflection per photodiode output) was calibrated by pressing the cantilever tip on an impressable substrate (glass slide in our case) so that the cantilever deflection Δ*d* equals the *z*-motor displacement Δ*z* (readily controlled and recorded) (fig. S7A). The calibrated sensitivity was 53.87 nm V^−1^ in our case by averaging sensitivity values from five different spots. The spring constant was then calibrated by fitting the thermal noise spectrum in the software and determined to be 0.484 N m^−1^. The *z*-motor approaches the sample at a speed of 1 μm s^−1^. The cantilever extension set point was set to 10 nN, at which point the approaching stops and *z*-motor moves away from the sample to retract the cantilever. For each SF block, a central area of 10 μm by 10 μm was indented using the force mapping mode with 4 × 4 points. Before the cantilever moved to the next point, after full retraction of the current sampling point, a 10-s waiting time was executed to allow the indented soft gel to recover. The Hertz model was used to fit the extension curve to obtain the Young’s modulus, *E*, of the SF blocks. The force baseline and the contact point were also fitted parameters. The fitting range was determined by plotting the indentation δ-fitted *E* curve to identify the plateau position (fig. S7B). In the case of the SF blocks printed with a laser power of 60%, the fitted *E* values reached a plateau after an indentation depth of 350 nm. The fitting range was set to 500 nm to fit all curves of these SF blocks. Each fitting result was checked for fitting quality; poorly fitted or abnormal curves were discarded. The *E* values in the central 50% range for each SF block were used to calculate the mean and SD.

To validate the used AFM measurement parameters and the fitting method, we performed bulk mechanical tests because the two methods (microscopic and macroscopic) should give theoretically the same results for the same material. We used a reference agarose gel to validate the measurements because it is impossible to 2PP-print a bulk-sized SF block, which should take days and during which the SF precursor solution would have completely cross-linked. Using the same AFM cantilever and the same testing procedure, the obtained modulus of a 0.8% agarose gel thin film was determined to be 15.1 ± 1.3 kPa. Bulk cylindrical (3 mm thick and 8 mm in diameter) 0.8% agarose gel samples were characterized by compression (TA Discovery Hybrid HR-3 rheometer with a 20-mm top plate), and the measured modulus was 14.8 ± 2.1 kPa, which fits well with the AFM results.

### SF rheological analysis

The oscillation rheological measurement was conducted with bulk SF hydrogel samples using a TA Discovery Hybrid HR-3 rheometer. The silk hydrogel precursor had the same composition as that for fabricating the MAC, but instead of 2PP-printed, it was cross-linked under 365-nm UV light for 30 min in a cylindrical PDMS mold with a diameter of 20 mm and depth of 3 mm. The mold was covered with a transparent glass slide during the UV exposure to prevent solution evaporation. The cross-linked SF hydrogel was immersed in DI water for 1 hour before the test. For the rheometer setup, we used a parallel top plate with a diameter of 20 mm. The SF hydrogel was placed on the bottom plate, and the top plate was slowly lowered to bring full contact with the hydrogel but without significant pressing by limiting the axial force below 0.05 N. We first conducted strain amplitude sweep at 6.28 rad s^−1^ (1 Hz) to identify the linear viscoelastic region to be below 0.06%, in which region the hydrogel network structure is not irreversibly broken. Then, we did a frequency sweep in the range of 0.1 to 620 rad s^−1^ under a constant 0.01% strain.

### MAC beam swelling characterization

The swelling of the MAC beams at room temperature was characterized by measuring their lengths using the inverted optical microscope of the 2PP system with the 63× oil-immersion objective. The tested samples were printed with various laser powers under a constant scanning speed of 10,000 μm s^−1^. At least five MAC were fabricated and measured for each laser power. The lengths of the MAC beams were measured before and after developing with DI water and PBS, respectively, over a period of 48 hours in a sealed microfluidic chamber.

The qualitative swelling of the MAC beams under elevated temperature was tested using a digitally controlled microscope heating stage (Pola vis P390) under a Zeiss Axio Imager M2 microscope with a 20× objective. The tested samples were printed with various laser powers under a constant scanning speed of 10,000 μm s^−1^. At least five MAC were fabricated and measured for each laser power. The as-fabricated samples were developed with DI water and soaked in DI water for 24 hours to reach hydrogel equilibrium before testing. The temperature was ramped from room temperature to 90°C with a 10°C step. At each temperature, the samples were retained for 10 min to reach temperature equilibrium. The qualitative swelling of SF bridges fabricated with various laser powers and scanning speeds were also tested using the same method. The quantitative swelling of the MAC beams before and after baking at 60°C were measured using the optical microscope of the 2PP system with the 63× oil-immersion objective. The baking was done using a commercial hot plate for 10 min, and the lengths of the MAC beams after baking were measured after the samples cooled down to room temperature.

### MAC bending characterization

The bending performance of the MAC was characterized using the inverted optical microscope of the 2PP system with the 63× oil-immersion objective. The tested samples were printed with various laser powers under a constant scanning speed of 10,000 μm s^−1^. At least five MAC were fabricated and measured for each laser power. The maximum bending angle θ of the MAC beating at 0.5 Hz in DI water or PBS was measured from recorded videos of 100 frames per second (fps).

### Biocompatibility and biodegradability tests

Biocompatibility tests of the MAC were conducted with human fibroblast cells, CRL-2522. The cells were obtained from the American Type Culture Collection (Rockville, MD, USA). The cells were precultured in Gibco Minimum Essential Media supplemented with 10% v/v fetal bovine serum, penicillin (50 UI ml^−1^), and streptomycin (50 μg ml^−1^) in a humidified, 37°C, 5% CO_2_ polystyrene cell culture flask. For the biocompatibility experiments, glass slides containing the MAC were sterilized and then incubated with fibronectin (50 μg ml^−1^) for 2 hours at 37°C. To ensure a thorough sterilization, the PDMS microfluidic chips were removed before sterilization. The treated slides were then transferred to 35-mm cell culture flasks, and 0.5 × 10^6^ cells were seeded. After 96 hours, the viability of the cells was examined with the LIVE/DEAD Viability/Cytotoxicity Kit (Invitrogen, Thermo Fisher Scientific, Waltham, MA) using a fluorescence microscope (Eclipse Ti-E; Nikon, Tokyo, Japan). The tested MAC were printed with various laser powers under a constant scanning speed of 10,000 μm s^−1^. At least five MAC were fabricated and measured for each laser power.

The biodegradability test of the MAC was conducted at room temperature under a flow of protease XIV solution at 1 U ml^−1^ (protease from *Streptomyces griseus*, type XIV; Sigma-Aldrich) at a flow rate of 2 μl min^−1^ controlled by a syringe pump (KD Scientific Inc., Holliston, MA). Three MAC printed with a laser power of 60% and a scanning speed of 10,000 μm s^−1^ were tested under an inverted microscope (Zeiss Axio Observer A1, Carl Zeiss, Oberkochen, Germany). A video was taken at a frame rate of 10 fps. Both bright-field and fluorescent microscopic images were taken before and after the experiments. The used MAC were developed with DI water and soaked in DI water for 24 hours before the biodegradable test.

### Characterization of the MAC motion and the generated fluidic flow

The actuation experiments of the MAC were performed using equilibrated 1-day-old samples after developing with DI water. The normal MAC and F-MAC were actuated under an in-plane uniform rotating magnetic field of 3 and 6 mT, respectively, produced by a custom-built four-coil electromagnetic setup. A high-speed camera (Phantom Miro M310, Vision Research, Ametek Inc.) connected to the Zeiss Axio Observer A1 inverted microscope was used to record the MAC motion at a frame rate of 500 fps. The MAC tip speed was analyzed with the Manual Tracking add-on in ImageJ. The time-lapse images shown in Fig. 3 and fig. S13 were composed by stacking the high-speed images using the Z Project function in ImageJ.

For fluid transporting experiments, the MAC were developed with DI water seeded with 2-μm tracer particles at a flow rate of 0.5 μl min^−1^ using a syringe pump (KD Scientific Inc., Holliston, MA). The fresh normal MAC and F-MAC were actuated at 5 Hz under an in-plane uniform rotating magnetic field of 3 and 6 mT, respectively. The generated flows at different height levels above the glass substrate were recorded by the high-speed camera at a frame rate of 50 fps. The tracking of the tracer particles was performed using an in-house Python script with the Trackpy package (*66*), which was validated using the Manual Tracking add-on in ImageJ. The time-lapse images shown in Figs. 5 and 6 were composed by stacking the high-speed images at a 10-frame interval using the Z Project function in ImageJ. Note that the tracked trajectories of the tracer particles in the particle tracking images shown in Figs. 5 and 6 are not the results of the tracer particles moving completely for 30 or 60 s because some particles are going out of focus and unable to be tracked and some particles are entering and exiting the observed area now and then. The flow speeds shown in Figs. 5D and 6 (B and D) were obtained by averaging the velocities of the tracer particles within a surface area of 300 μm by 400 μm over a time period of 30 s, within a surface area of 400 μm by 400 μm over a time period of 60 s, and within a surface area of 375 μm by 500 μm over a time period of 30 s, respectively.

### Mechanics of metachronal waves

Each cilium within a MAC array is an independent micromotor subject to a uniform rotating magnetic field **B**(*t*). The phases of these micromotors can be represented by their body slope angles θ*_i_*(*y*, *t*) at time *t*. According to the Euler-Bernoulli beam theory (*20*), the moment-balancing equations for two neighboring cilia (cilium *i* and *i* + 1) are as follows

$$-EI\frac{\partial^{2}\theta_{i}(y,t)}{\partial y^{2}}=\tau_{i}^{\mathrm{mag}}[\theta_{i}(y,t)]+[F_{h}(y,t)\cos\theta_{i}(y,t)-F_{v}(y,t)\sin\theta_{i}(y,t)]$$ (1)

$$-EI\frac{\partial^{2}\theta_{i+1}(y,t)}{\partial y^{2}}=\tau_{i+1}^{\mathrm{mag}}[\theta_{i+1}(y,t)]+[F_{h}(y,t)\cos\theta_{i+1}(y,t)-F_{v}(y,t)\sin\theta_{i+1}(y,t)]$$ (2)

where *E* is the Young’s modulus, *I* is the second moment of inertia of the cross section of the SF beam, **F**(*y*, *t*) = [*F_h_ F_v_*]*^T^* is the internal force of the cilium, and ${\text{τ}}_{i}^{\mathrm{mag}}$ and ${\text{τ}}_{i+1}^{\mathrm{mag}}$are the magnetic torques applied on the *i*-th and (*i* + 1)–th cilia, which can be obtained by

$${\text{τ}}_{i}^{\mathrm{mag}}=m_{i}\times B(t)$$ (3)

$${\text{τ}}_{i+1}^{\mathrm{mag}}=m_{i+1}\times B(t)$$ (4)

where **m***_i_* and **m**_*i*+1_ are the magnetization of the *i*-th and (*i* + 1)–th cilia. **m***_i_* and **m**_*i*+1_ have the same magnitude but with an orientation shift of Δψ. This orientation difference results in the temporal differences in the time-varying and spatially distributed external magnetic torque τ^mag^, which leads to the phase difference ΔΦ in the beating motion of neighboring cilia, creating metachronal waves.

### Numerical simulations of the MAC motion and the generated fluidic flow

We used a 3D computational fluid dynamics (CFD) model to simulate the MAC (both normal MAC and F-MAC) motion and the generated fluidic flow (*54*). This model accounts for the fully coupled bidirectional solid-liquid interactions between the MAC and the fluid and can accurately describe the magnetic torque imposed on the FePt JMPs, the deformation of the SF beam, the additional flag-shaped structure, and the deformation-induced fluid flow at low *Re*. The computational framework is briefly summarized here. At the low *Re*, inertia can be neglected so that we can model the fluid around the MAC using the Stokes equation

$$\mu \nabla^{2}u+\nabla p=0$$ (5)

with μ as the fluid viscosity, ∇**p** as the pressure gradient, supplemented by the continuity equation describing incompressible flow, and ∇ · **u** = 0. The drag forces on the MAC surface were regarded as a distribution of point forces **f**(**r**). The fluid velocity **u***^f^* at a point due to these point forces **f**(**r**) exerted on the fluid by the MAC can be written as

$$u^{f}(r)=G[r-r^{\prime },h(r^{\prime })]\;f(r^{\prime })$$ (6)

where **h**(**r**′) is the distance between the point force and the bottom surface and **G**[**r−r**′, **h**(**r**′)] is the Green’s function for a point force acting in a Stokes flow near a no-slip boundary (*67*). These point forces were assumed to be distributed over the surface of the MAC as a traction **t**(**r**′), which was linear over each triangular surface element resulting in the fluid velocity

$$u^{f}(r)=\sum_{j=1}^{\mathrm{nelm}}\int G[r-r^{j},h(r^{j})]t(r^{j})dS_{j}$$ (7)

Here, nelm is the total number of MAC surface elements. Because Eq. 7 holds at every point in the fluid, it also holds at every node *i* on the MAC surface **u***^c^*(**r***^i^*) through **u***^c^* = **Gt**. Therefore, the surface traction **t** exerted by the MAC on the fluid because of its velocity **u***^c^* can be calculated from **t** = **G**^−1^**u***^c^*. The surface traction was then imposed as external forces to the solid mechanics model of the MAC. The solid and fluid mechanics models were coupled by the no-slip boundary. The magnetic couples **τ** were due to the MAC magnetization **m** and the externally applied rotating magnetic field (**B**) by using **τ** = **m** × **B**. More details on the numerical model can be found in our previous work (*54*, *63*). The geometry and the magnetic and mechanical properties of the MAC were taken directly from the experimental results.

### Statistical analysis

The SDs are indicated in the figure captions.
