# Supplementary material for: 3D-printed micrometer-scale wireless magnetic cilia with metachronal programmability
Source: Sci Adv. 2023 Mar 22;9(12):eadf9462. doi: 10.1126/sciadv.adf9462 (PMC7614626; doi:10.1126/sciadv.adf9462)
Supplement: Supplementary file 1 — Supplementary Text Figs. S1 to S15 Legends for movies S1 to S13 [file sciadv.adf9462_sm.pdf]

Science Advances
AAAS

Supplementary Materials for

3D-printed micrometer-scale wireless magnetic cilia with metachronal programmability

Shuaizhong Zhang *et al.*

Corresponding author: Metin Sitti, sitti@is.mpg.de

Sci. Adv. **9**, eadf9462 (2023)
DOI: 10.1126/sciadv.adf9462

The PDF file includes:

Supplementary Text
Figs. S1 to S15
Legends for movies S1 to S13

Other Supplementary Material for this manuscript includes the following:

Movies S1 to S13

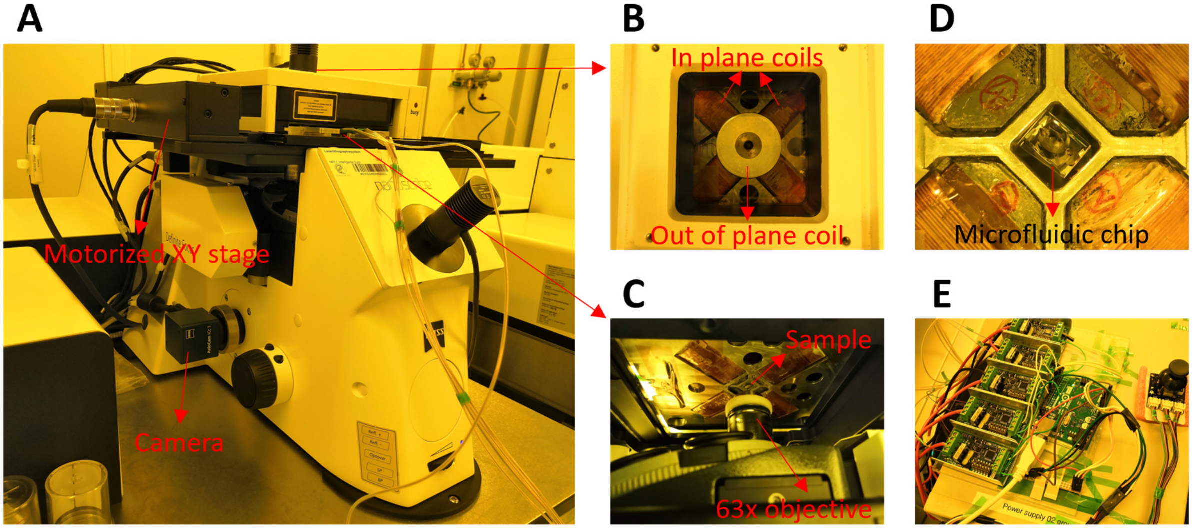

Fig. S1 Photos of the experimental setup for fabricating the magnetic artificial cilia (MAC). (**A**) Two photon polymerization system (2PP, Photonic Professional, Nanoscribe GmbH, Germany) integrated with a 5-coil electromagnetic setup. (**B**) Top view of the coil setup embedded in the 2PP system. It is able to apply a magnetic field up to 18 mT at the center of the working space. (**C**) Bottom view of the coil setup, showing that a sample is mounted by rubber glue (Marabu Fixogum, Article number: 283-290110) at the center of the coil setup and that a 63x oil immersion objective is used for direct laser printing and sample observation. (**D**) Top view of the sample consisting of a microfluidic chip (Fig. S3) bonded to a glass substrate (12 mm × 12 mm). The microfluidic chip is sealed with a thin PDMS film (200 µm thick), producing a closed environment to prevent evaporation of SF. (**E**) A custom electronic board connected with a joystick to control five independent electromagnets. Photo credit: Shuaizhong Zhang, Max Planck Institute for Intelligent Systems.

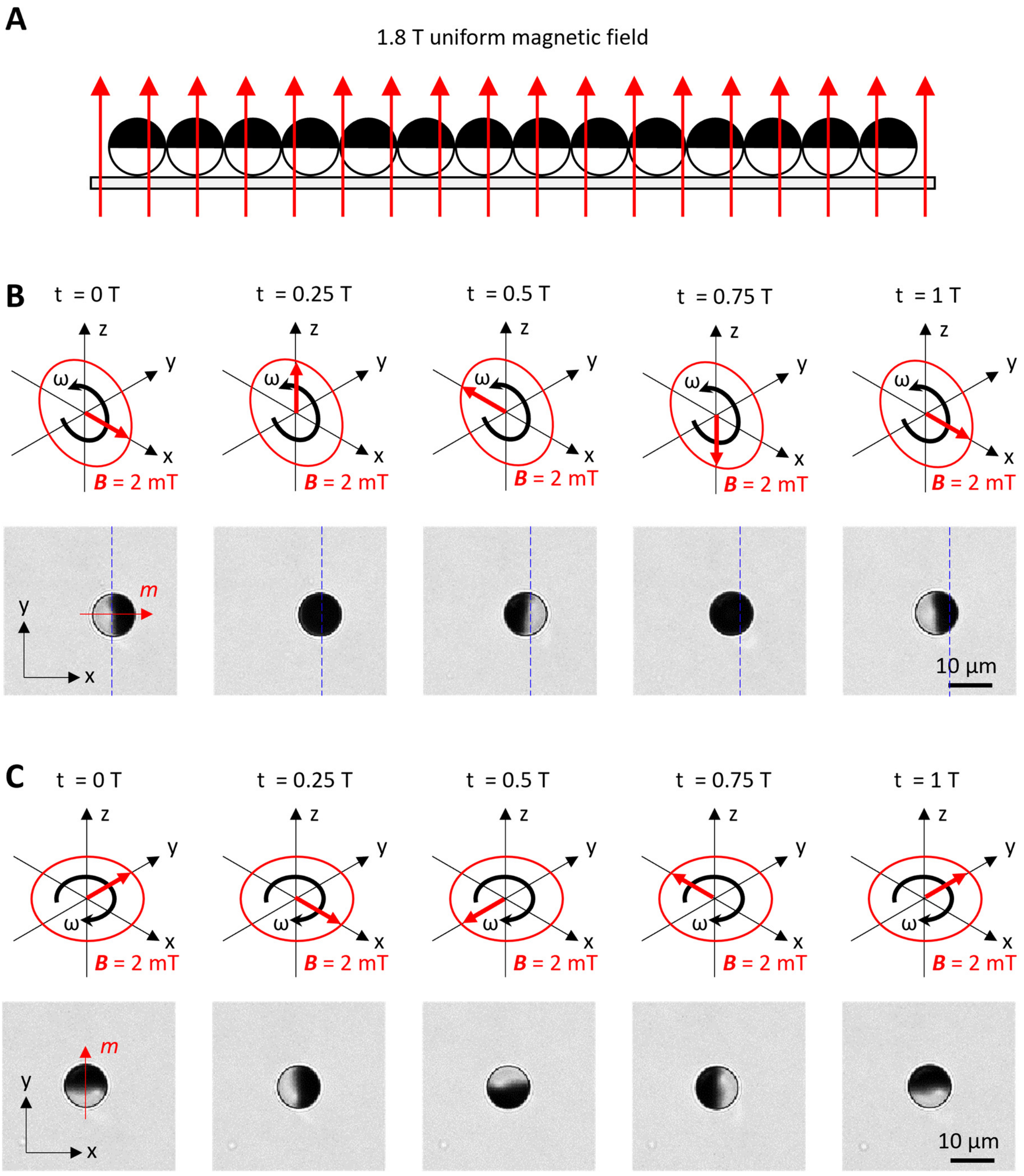

Fig. S2 Magnetic positioning and orientating of a FePt-JMP under an applied uniform rotating magnetic field. (**A**) Magnetization of the FePt-JMPs along the normal direction of the interface between the FePt half and the silica half under a 1.8 T uniform magnetic field. (**B**) Out-of-plane (x-z) rotation of a FePt-JMP under a rotating magnetic field ***B*** (2 mT) at 0.5 Hz, resulting in surface rolling of the FePt-JMP, which leads to the its positioning. ***m*** indicates the magnetic moment of the FePt-JMP, and the dashed blue lines indicates the original location of the FePt-JMP. (**C**) In-plane (x-y) rotation of a FePt-JMP under a rotating magnetic field ***B*** (2 mT) at 0.5 Hz. The FePt-JMP aligns its magnetization direction with the external field, resulting in in-plane rotation, which leads to its orientation.

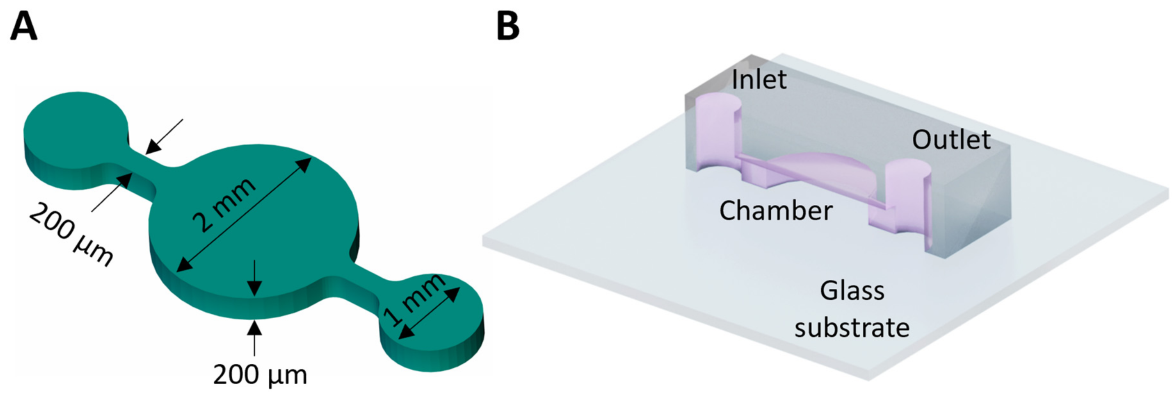

Fig. S3 Design of the used PDMS microfluidic chip. (**A**) The geometry of the microfluidic channel. (**B**) Cross-sectional view of the microfluidic chip.

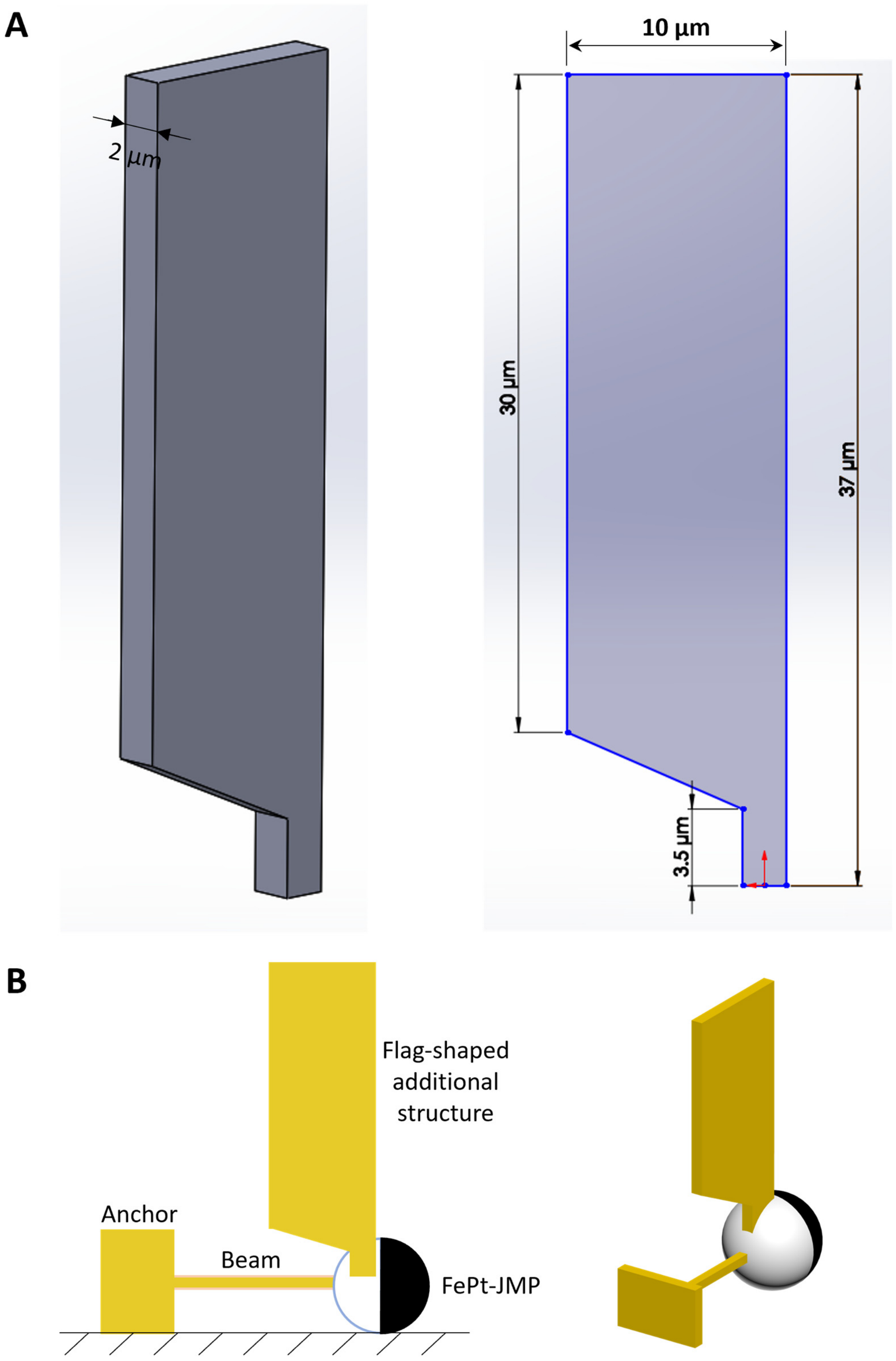

Fig. S4 Design of the 3D-printed flag-shaped additional structure on the FePt JMP. (**A**) Oblique and side view of the flag-shaped structure showing its geometry. (**B**) Cross-sectional and oblique view of the cilium with the flag-shaped structure showing the relative position of the flag.

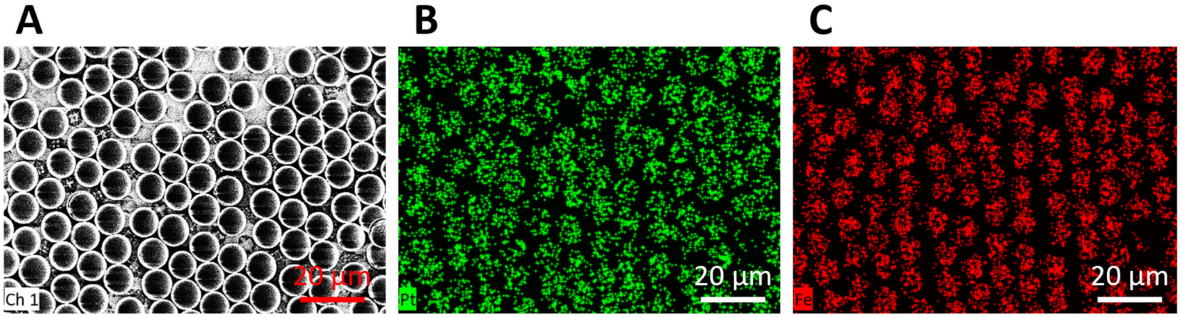

Fig. S5 SEM image and energy dispersive x-ray spectroscopy (EDX) scan of the 60 nm-thick FePt JMPs. (**A**) SEM image of the monolayered FePt JMPs used for EDX scan. (**B**,**C**) EDX map of Pt and Fe, showing homogeneous distribution on the surface of microparticles.

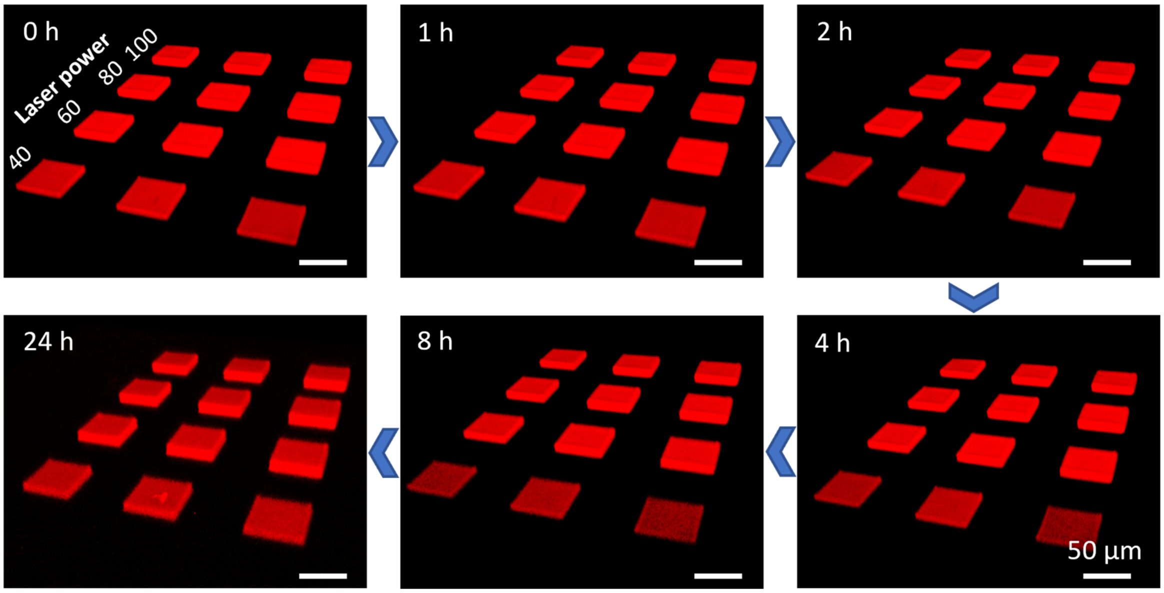

Fig. S6 Confocal microscopy time-lapse images of the 2PP-printed SF microstructures. Time-lapse 3D images of the 3D-printed SF microstructures with different laser powers (40%, 60%, 80%, and 100%) show their swelling behaviors over 24 hours.

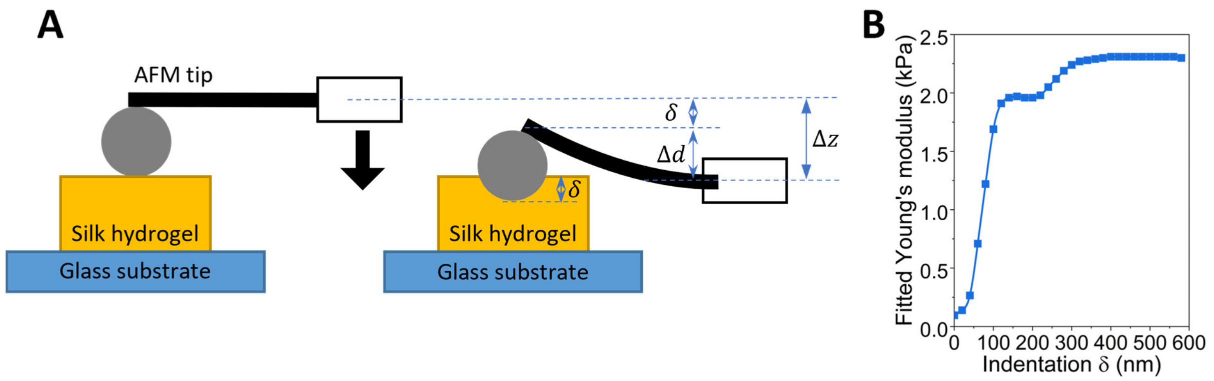

Fig. S7 Characterization of the Young's modulus of the 2PP-printed SF microstructures using AFM nanoindentation. (**A**) Schematics of the measuring mechanism. (**B**) The measured Young's modulus as a function of the AFM tip indentation.

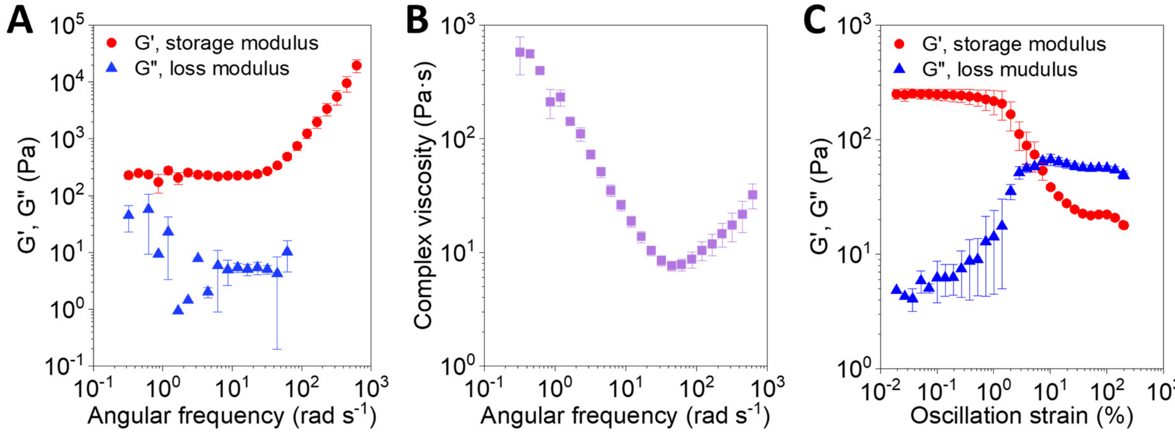

Fig. S8 Characterization of the rheological properties of bulk SF hydrogels using dynamic oscillation measurement. (**A**) Storage modulus and loss modulus as a function of oscillation frequencies at strain 0.01%. (**B**) Complex viscosity as a function of oscillation frequencies. (**C**) Storage modulus and loss modulus as a function of oscillation strain at frequency 6.28 rad s^{-1}. Error bars indicate SDs for n = 3 measurements.

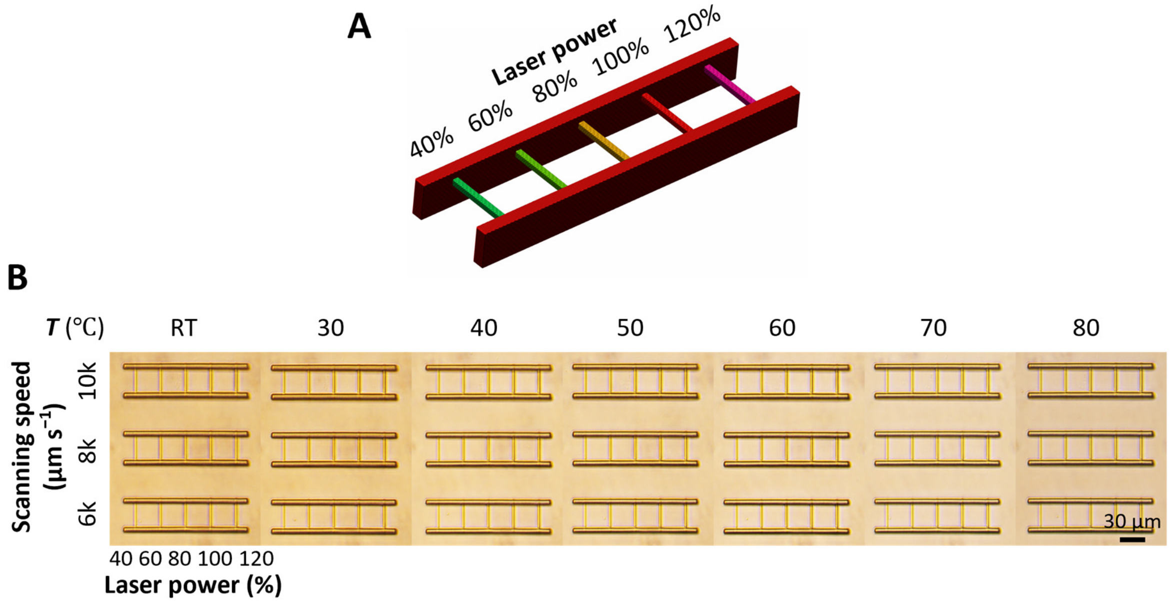

Fig. S9 Mechanical stability of the 2PP-printed SF microstructures under elevated temperatures. (**A**) Schematic of the design of the SF microstructures. The SF beams have a width, height and length of 2 μm, 2 μm and 30 μm, respectively. (**B**) Top-view microscopy images of the SF microstructures under elevated temperature, showing no visible shape change even at 80 °C. The heating duration for each temperature is 10 minutes.

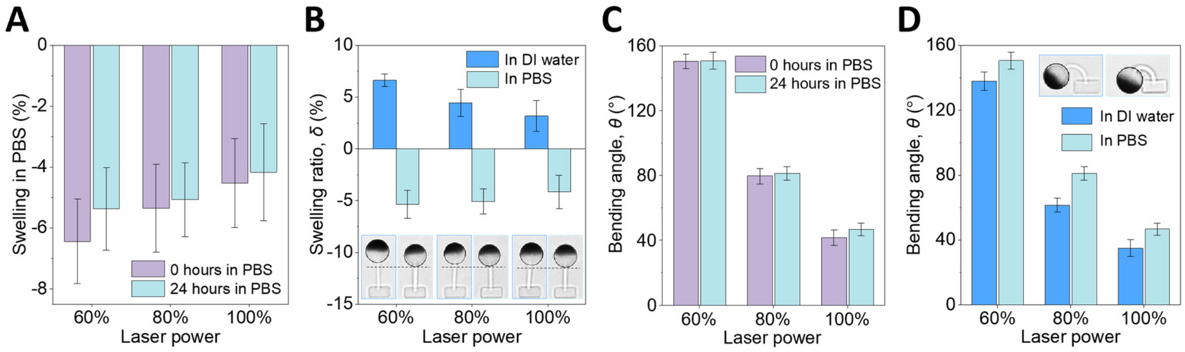

Fig. S10 Mechanical stability of the MAC in PBS. (**A**) The linear swelling behavior of the MAC in PBS over time, showing decreased shrinkage over time. (**B**) Comparison of the linear swelling behavior between the MAC developed with DI water and PBS, showing contradictory swelling phenomena. (**C**) The maximal bending angle θ of the MAC in PBS over time, showing no obvious change. (**D**) Comparison of the maximal bending angle θ between the MAC developed with DI water and PBS, showing approximately 30% larger bending angles in PBS than in DI water, except for the MAC printed with a laser power of 60% which only show 10% lager bending in PBS because of the reach of the maximum value. Error bars indicate SDs for $n \geqslant 5$ measurements.

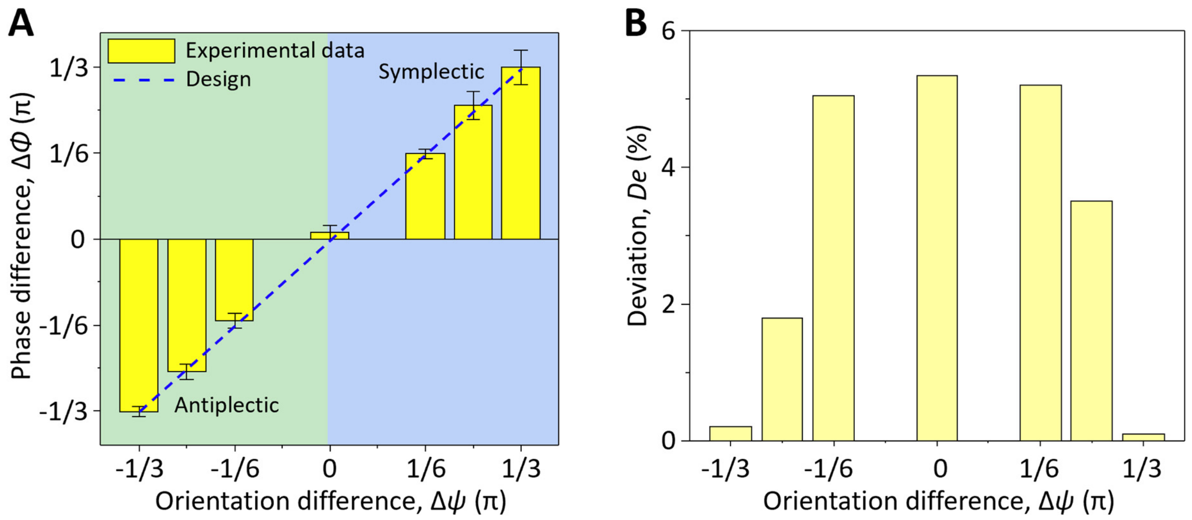

Fig. S11 Quantitative analysis of the experimental phase difference, $\Delta\Phi$. (**A**) Measured $\Delta\Phi$ as a function of the designed phase difference, $\Delta\psi$. (**B**) The deviation, *De*, between $\Delta\Phi$ and $\Delta\psi$, which is defined by $De = \begin{cases} \frac{\Delta\psi - \Delta\Phi}{\Delta\psi}, \Delta\psi \neq 0 \\ |\Delta\psi - \Delta\Phi|, \Delta\psi = 0 \end{cases}$. All the cilia mentioned here were printed with a laser power of 60% at a scanning speed of 10000 μm s^{-1}. Error bars indicate SDs for $n \geq 5$ measurements.

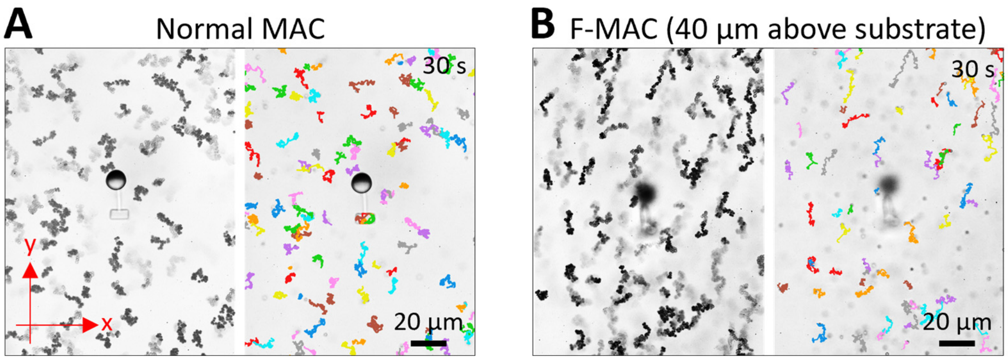

Fig. S12 Fluid transporting capability of a single artificial cilium. Time-lapse (left) and particle-tracking (right) images of one static control experiment over a period of 30 s for (**A**) A normal cilium and (**B**) a F-MAC. The overlapping images are composed of 150 consecutive images of 30-second videos. The observation layer for panels **A** and **B** is 10 µm and 40 µm above the substrate, respectively.

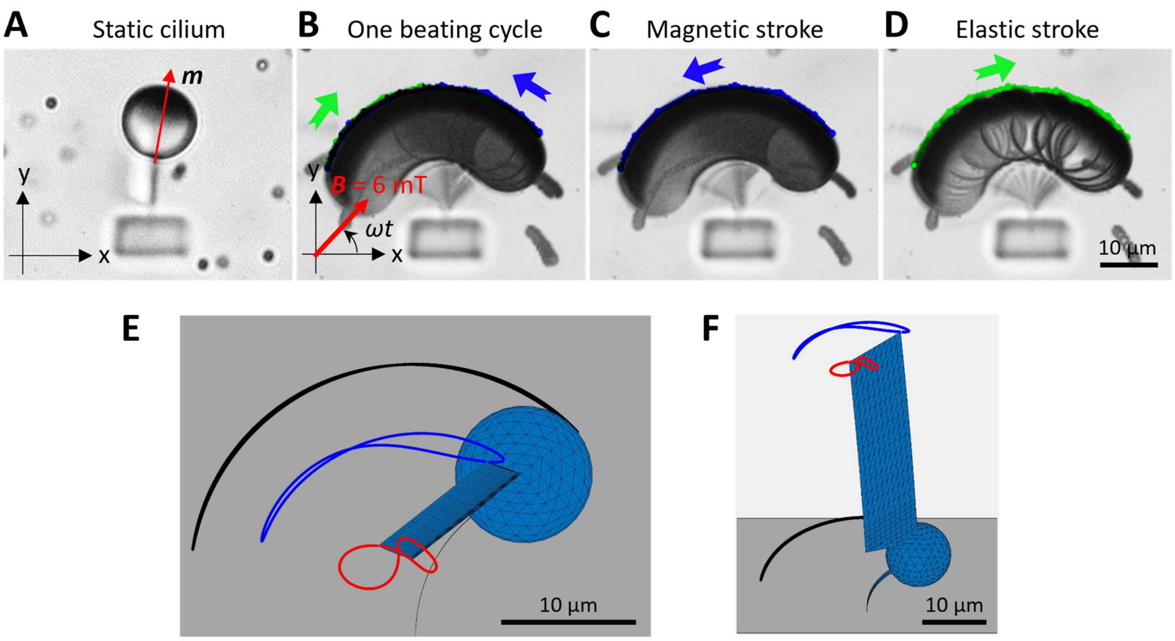

Fig. S13 F-MAC motion at 5 Hz under a uniform magnetic field of 6 mT rotating in the counterclockwise direction. (A) Bottom-view microscopic image of one static F-MAC with the red arrow indicating the magnetization direction of the FePt-JMP. (**B-D**) Time-lapse images of the F-MAC motion during one beating cycle, showing a 2D reciprocal motion. The blue and green arrows indicate the direction of the magnetic stroke and the elastic stroke, respectively. The images are composed of image sequences with an identical time interval of 0.002 s. (**E**,**F**) Top-view (**E**) and oblique-view (**F**) images of the simulated F-MAC motion during one beating cycle. The black, blue and red lines indicate the trajectories of the FePt-JMP and the two upper corners of the SF flag, respectively, showing that the F-MAC body performs a reciprocal motion while the flag undergoes a nonreciprocal motion.

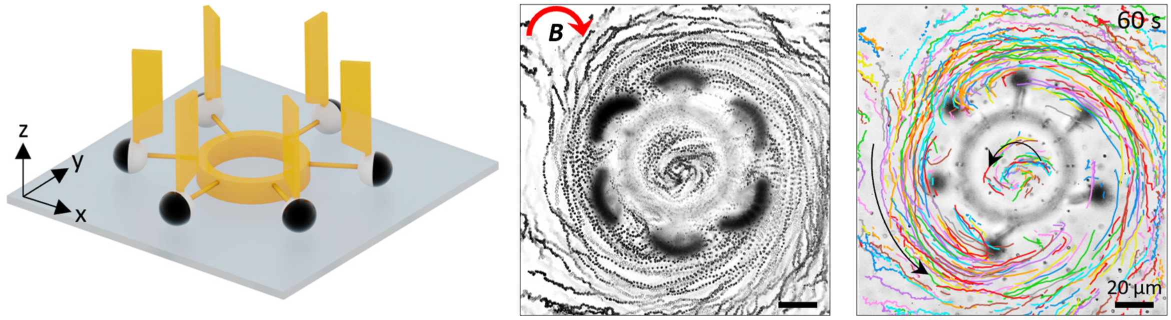

Fig. S14 Schematic of the circular F-MAC arrangement (left), and time-lapse (middle) and particle-tracking (right) images of the generated flow over a period of 60 s composed of 300 images. The red arrow marks the rotating direction of a 6 mT uniform magnetic field and the black arrows indicate the flow direction (see Movie S13).

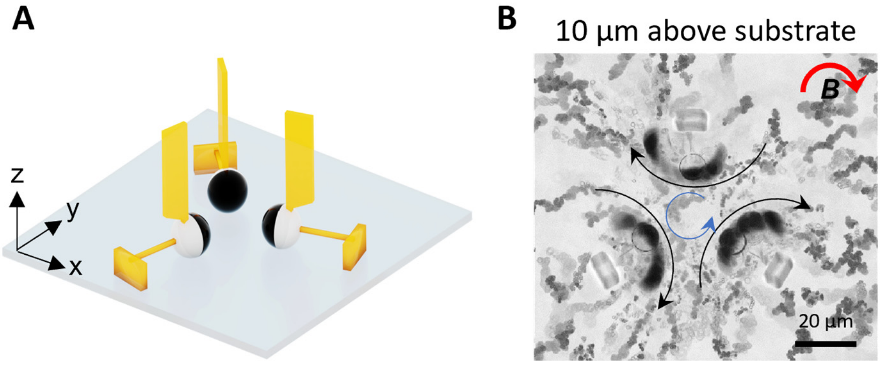

Fig. S15 Programmable flow patterns. (**A**) Schematic of the triangularly configured 3-F-MAC array. (**B**) Time lapse images of the fluid transporting experiments, showing three curved flow paths as well as an anti-clockwise circular flow, i.e. an inward mixing flow. The observation level is 10 μm above the substrate. The time lapse images are composed of 100 images over a 20-second period. The black and blue arrows indicate the flow directions. The red arrow marks the rotating direction of a 6 mT uniform magnetic field at 5 Hz.

Supplementary Note 1. Mechanics for the nonreciprocal motion and the definition of the swept area of the F-MAC

Nonreciprocal motion indicates that there is a difference in the swept area by the cilia during their two beating strokes (i.e. effective/power stroke and recovery stroke). In terms of a 2D whip-like motion, the swept area difference derives from the bending difference of the cilia during the two beating strokes. For an active cilium as shown in ref. 20, the bending difference is resulted from the encoded magnetic profile of the cilia themselves. While, in our case, the bending difference is attributed to the passive silk fibroin appendages (the so-called SF flag), which is observed experimentally and confirmed numerically (Fig 5 and Fig. S13). The mechanics underlying this difference is simply because much larger hydrodynamic drag forces exert on the SF flag during the elastic stroke than during the magnetic stroke. This is due to the much faster moving speed of the SF flag during the elastic stroke than during the magnetic stroke as observed and carefully analyzed experimentally and confirmed numerically (Fig 5 and Fig. S13). According to the drag equation

$$F_d = \frac{1}{2}\rho u^2 c_d A, \tag{S1}$$

where $F_d$is the hydrodynamic drag force exerted on the SF flag, ρ is the mass density of the fluid, u is the flow velocity relative to the SF flag, c_d is the drag coefficient, and A is the reference area; a larger moving speed results in a larger hydrodynamic drag on the SF flag. And according to the cantilever beam deflection equation

$$\delta_B = \frac{F_d L^3}{3EI}, \tag{S2}$$

where δ_B is the induced deflection of the SF flag, L is the height of the SF flag, E is the Young's modulus of the SF flag, and I is the area moment of inertia of cross section of the SF flag; the larger hydrodynamic drag leads to a larger bending deformation of the SF flag. In summary, a lager moving speed of the SF flag contributes to a larger bending deformation, and thus a nonreciprocal motion is induced.

More detailed mathematical model of the driving forces for the nonreciprocal motion can be found in the description of the numerical simulations in the **Materials and Methods** section.

The swept area of a 2D whip-like motion represents the area of the ciliary trajectories projected onto the motion plane (in our case, the *xy* plane) during one beating cycle, which is commonly used by the cilia community (refs. 1, 2, 18, 20, 30, 62 and 66). We also employed this definition to define the swept area of our cilia. However, in our case where our F-MAC have a 3D geometry, the swept area have two components: (1) the area swept by the cilia body (the Janus particles attached with the SF beams), which is projected onto the *xy* plane; during one beating cycle, the swept area of elastic and magnetic strokes cancels out and no net swept area is induced; (2) the area swept by the SF appendage, i.e. the SF flag, which is projected onto both the *xz* and *yz* planes as the flag undergoes a 3D trajectory. The former induces no net swept area because of the reciprocal motion, while the latter results in a net swept area as observed experimentally and confirmed numerically.

Legends for movies S1-S12

Movie S1. Magnetic actuation and control of FePt-Janus microparticles.

Movie S2. Fabrication process of a cilium with a flag-shaped additional structure.

Movie S3. FePt-JMPs aggregation and separation.

Movie S4. Biodegradability of the MAC.

Movie S5. Experimental and numerically simulated MAC motion under a uniform rotating magnetic field of 3 mT at 1 Hz.

Movie S6. Programmable metachrony of MAC arrays under a uniform rotating magnetic field of 3 mT at 0.5 Hz.

Movie S7. Metachrony of MAC arrays created by varying the mechanical properties of the SF beams.

Movie S8. Programmable metachrony of 2D MAC arrays under a uniform rotating magnetic field of 3 mT at 0.5 Hz.

Movie S9. Experimental and simulated F-MAC motion under a uniform rotating magnetic field.

Movie S10. Fluid transporting capability of a single F-MAC under a uniform rotating magnetic field of 6 mT at 5 Hz. Note that the particle tracking videos have a frame rate of 5 fps since they are extracted from the original videos (which have a frame rate of 50 fps) with a 10-frame interval to show more clearly the trajectories of the moving tracer particles.

Movie S11. F-MAC motion under a uniform rotating magnetic field of 6 mT at 100 Hz. The video shows that the beating frequency of the F-MAC is the same as the actuation frequency, meaning no stepping-out of the MAC motion.

Movie S12. Fluid transporting capability of metachronal F-MAC arrays under a uniform rotating magnetic field of 6 mT at 5 Hz. Note that the particle tracking videos have a frame rate of 5 fps since they are extracted from the original videos (which have a frame rate of 50 fps) with a 10-frame interval to show more clearly the trajectories of the moving tracer particles.

Movie S13. Circulating and mixing flows generated by a circular metachronal F-MAC array under a uniform rotating magnetic field of 6 mT at 5 Hz.
